# Mixed Response to Cancer Immunotherapy is Driven by Intratumor Heterogeneity and Differential Interlesion Immune Infiltration

**DOI:** 10.1158/2767-9764.CRC-22-0050

**Published:** 2022-07-28

**Authors:** Takao Morinaga, Takashi Inozume, Masahito Kawazu, Youki Ueda, Nicolas Sax, Kazuo Yamashita, Shusuke Kawashima, Joji Nagasaki, Toshihide Ueno, Jason Lin, Yuuki Ohara, Takeshi Kuwata, Hiroki Yukami, Akihito Kawazoe, Kohei Shitara, Akiko Honobe-Tabuchi, Takehiro Ohnuma, Tatsuyoshi Kawamura, Yoshiyasu Umeda, Yu Kawahara, Yasuhiro Nakamura, Yukiko Kiniwa, Ayako Morita, Eiki Ichihara, Katsuyuki Kiura, Tomohiro Enokida, Makoto Tahara, Yoshinori Hasegawa, Hiroyuki Mano, Yutaka Suzuki, Hiroyoshi Nishikawa, Yosuke Togashi

**Affiliations:** 1 Chiba Cancer Center, Research Institute, Chiba, Japan.; 2 Department of Dermatology, Graduate School of Medicine, Chiba University, Chiba, Japan.; 3 Department of Dermatology, University of Yamanashi, Yamanashi, Japan.; 4 Division of Cellular Signaling, National Cancer Center Research Institute, Tokyo, Japan.; 5 Department of Tumor Microenvironment, Okayama University Graduate School of Medicine Dentistry and Pharmaceutical Sciences, Okayama, Japan.; 6 KOTAI Biotechnologies Inc, Osaka, Japan.; 7 Department of Pathology, National Cancer Center Hospital East, Kashiwa, Japan.; 8 Department of Genetic Medicine and Services, National Cancer Center Hospital East, Kashiwa, Japan.; 9 Department of Gastroenterology and Gastrointestinal Oncology, National Cancer Center Hospital East, Kashiwa, Japan.; 10 Department of Dermatology and Plastic Surgery, Faculty of Life Sciences, Kumamoto University, Kumamoto, Japan.; 11 Department of Skin Oncology/Dermatology, Saitama Medical University International Medical Center, Saitama, Japan.; 12 Department of Dermatology, Shinshu University School of Medicine, Nagano, Japan.; 13 Department of Allergy and Respiratory Medicine, Okayama University Hospital, Okayama, Japan.; 14 Department of Head and Neck Medical Oncology, National Cancer Center Hospital East, Kashiwa, Japan.; 15 Department of Applied Genomics, Kazusa DNA Research Institute, Chiba, Japan.; 16 Department of Computational Biology and Medical Sciences, Graduate School of Frontier Sciences, The University of Tokyo, Chiba, Japan.; 17 Division of Cancer Immunology, Research Institute/Exploratory Oncology Research and Clinical Trial Center (EPOC), National Cancer Center, Tokyo/Kashiwa, Japan.; 18 Department of Immunology, Nagoya University Graduate School of Medicine, Nagoya, Japan.

## Abstract

Some patients experience mixed response to immunotherapy, whose biological mechanisms and clinical impact have been obscure. We obtained two tumor samples from lymph node (LN) metastatic lesions in a same patient. Whole exome sequencing for the both tumors and single-cell sequencing for the both tumor-infiltrating lymphocytes (TIL) demonstrated a significant difference in tumor clonality and TILs’ characteristics, especially exhausted T-cell clonotypes, although a close relationship between the tumor cell and T-cell clones were observed as a response of an overlapped exhausted T-cell clone to an overlapped neoantigen. To mimic the clinical setting, we generated a mouse model of several clones from a same tumor cell line. Similarly, differential tumor clones harbored distinct TILs, and one responded to programmed cell death protein 1 (PD-1) blockade but the other did not in this model. We further conducted cohort study (*n* = 503) treated with PD-1 blockade monotherapies to investigate the outcome of mixed response. Patients with mixed responses to PD-1 blockade had a poor prognosis in our cohort. Particularly, there were significant differences in both tumor and T-cell clones between the primary and LN lesions in a patient who experienced tumor response to anti–PD-1 mAb followed by disease progression in only LN metastasis. Our results underscore that intertumoral heterogeneity alters characteristics of TILs even in the same patient, leading to mixed response to immunotherapy and significant difference in the outcome.

**Significance:**

Several patients experience mixed responses to immunotherapies, but the biological mechanisms and clinical significance remain unclear. Our results from clinical and mouse studies underscore that intertumoral heterogeneity alters characteristics of TILs even in the same patient, leading to mixed response to immunotherapy and significant difference in the outcome.

## Introduction

The acquisition of immune escape mechanisms, including various immunosuppressive molecules and/or immunosuppressive cells, is essential for cancer development and progression and one such mechanism involves the induction of programmed death 1 (PD-1)/PD-1 ligands (1–5). PD-1, which interacts with PD-1 ligands, is primarily expressed following the activation of T cells and suppresses T cell function, causing T cells to fall into a dysfunctional state called exhaustion after chronic antigen stimulation (6–8). Because of this, PD-1 blockade therapies are effective against various types of cancer, such as malignant melanoma, lung cancer, gastric cancer, and head and neck cancer, via reinvigoration of such exhausted T cells in the tumor microenvironment (TME; refs. 2, 3, 9–14).

Although tumor-infiltrating CD8^+^ T cells have been reported to play an important role in PD-1 blockade–mediated antitumor immune response (2, 3, 15, 16), not all such T cells attack tumor cells, containing nonspecific bystander T cells (17, 18). Thus, such tumor-specific T cells are considered to be difficult to elucidate in the TME. Furthermore, because neoantigens derived from somatic mutations can be recognized as nonself and elicit strong immune responses, patients with high tumor mutational burden (TMB), who are expected to have high numbers of neoantigens, have been reported to respond better to cancer immunotherapies (19–22). Thus, tumor-specific and neoantigen-specific T cells, rather than just tumor-infiltrating CD8^+^ T cells, seem essential for immunotherapies.

In recent years, progress in single-cell sequencing has enabled its use in a wide range of research field, including cancer research, contributing our deep understanding of the TME (23–25). Several groups used this technology and have revealed that skewed T-cell clonotypes have a high expression of well-known exhaustion signature molecules such as PD-1, LAG-3, TIM-3, 4–1BB, CD39, and CD103, and such exhausted T-cell clonotypes are speculated to attack tumor cells directly (tumor-specific T cells; refs. 26, 27). Recently, we analyzed tumor-infiltrating T cells using single-cell sequencing from patients melanoma treated with an anti–PD-1 mAb. In this study, the skewed exhausted T-cell clonotypes actually responded to autologous tumor cell lines and neoantigens derived from somatic mutations (28).

Despite great advances in cancer therapy, tumor heterogeneity continues to be a barrier for the successful treatment in not only cytotoxic chemotherapies and molecular targeted therapies but also immunotherapies (29–32). Particularly, neoantigen heterogeneity in tumor cells could induce resistance to immunotherapies, suggesting the importance of immune-cell heterogeneity in the TME (31, 32). Here, we obtained two tumor samples from lymph node (LN) metastatic lesions (LN1 and LN2) in the same patient. While they were located next to each other, whole-exome sequencing (WES) for tumors and single-cell sequencing for the TME showed significant differences in both tumor cell and exhausted T-cell clones. In addition, we created different single tumor cell clones from a same mouse tumor cell line. One of them responded to PD-1 blockade with an inflamed TME, but the other failed with a noninflamed TME, similar to a mixed response. Clinically, patients with a mixed response to immunotherapies had a poor prognosis in various cancer types. These findings suggest that mixed response to immunotherapies can be related to tumor and immune cell heterogeneities, leading to a poor prognosis.

## Materials and Methods

### Patients

**FIGURE 1:**
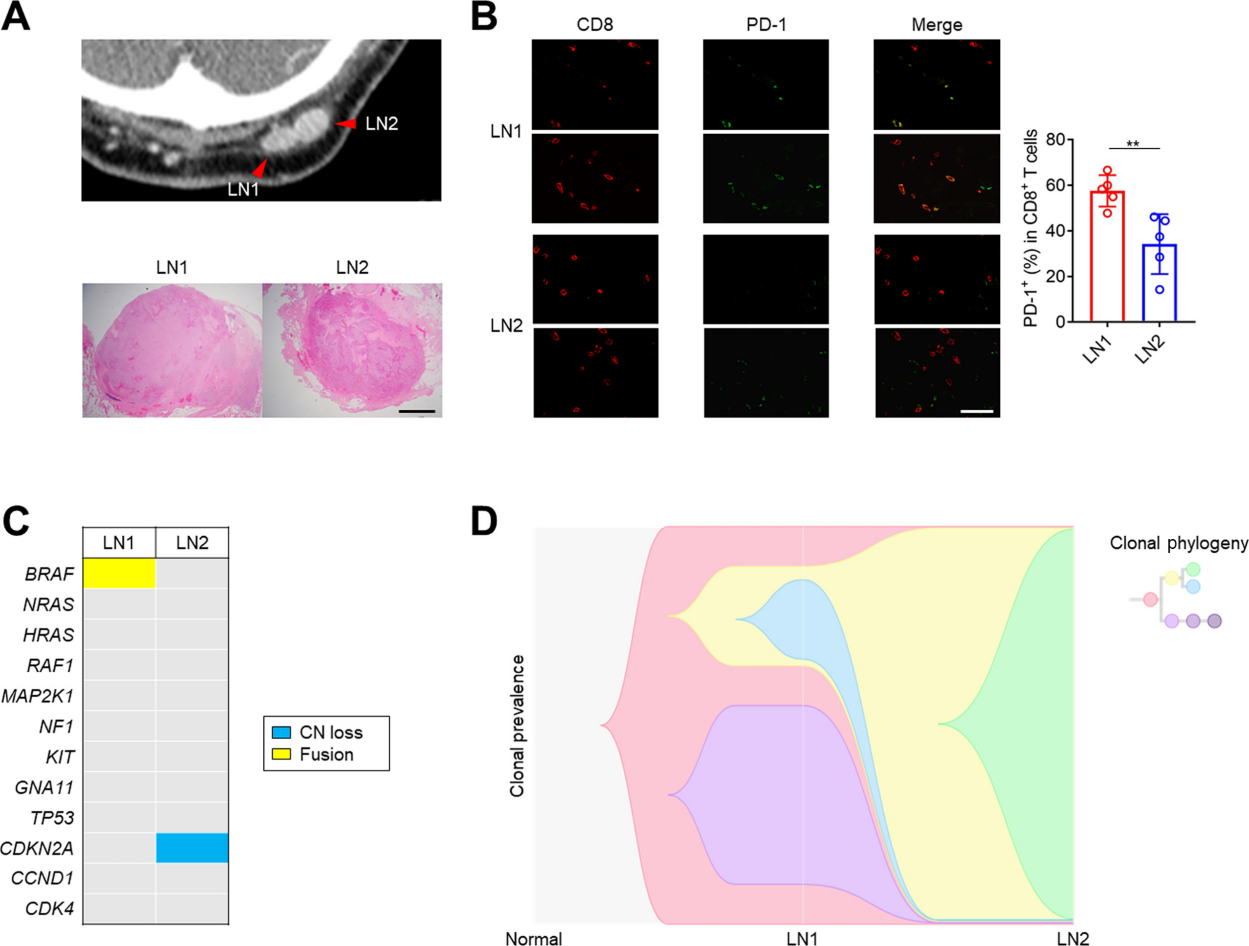
There were different tumor cell clones between LN1 and LN2. **A,** CT and the pathology of a male in his late 60s. Before the initiation of treatment, LN1 and LN2 were located next to each other (arrows), which were subsequently resected surgically. Computed tomography (top) and HE staining (bottom) are presented. Scale bars, 2 mm. **B,** IHC. FFPE sections (3 μm) from LN1 and LN2 were stained. Representative CD8 and PD-1 double staining and the quantified summary are presented. Scale bars, 50 μm. **C,** Representative driver gene alterations. Extracted DNA from LN1 and LN2 were sequenced and representative driver gene alterations are presented. CN, copy number. **D,** Clonal evolution of tumor cells. The cellular prevalence of clones carrying individual nonsynonymous mutations in LN1 and LN2 was determined using PyClone. The determined cellular prevalence was used as input, and the phylogenetic relationships of clones were inferred with LICHeE. The means and SDs are shown. A *t* test was used to calculate statistical significance in **B**. **, *P* < 0.01.

A male in his late 60s with advanced melanoma underwent surgical resection and received anti–PD-1 mAb at Yamanashi University Hospital. The metastatic lesion was resected *en bloc* and found to be comprised of two lesions separated by connective tissue from which LN1 and LN2 samples were separately obtained (Fig. 1A). Each tumor-infiltrating lymphocytes (TIL) and DNA extracted from resected samples were used for WES and single-cell sequencing. A male in his middle 60s with advanced gastric cancer received anti–PD-1 mAb as third-line therapy at National Cancer Center Hospital East (Supplementary Fig. S1). Autopsy samples were used for WES and TCR sequencing. We obtained written informed consent for analyses. In addition, clinical data of various cancer patients who received PD-1 blockade monotherapies without any cytotoxic chemotherapies were analyzed to evaluate mixed responses in this study (Supplementary Tables S1–4). The protocols for these studies were approved by the appropriate institutional review board and ethics committees at the Yamanashi University Hospital, Chiba University Hospital, Shinshu University Hospital, Saitama Medical University International Medical Center, Okayama University Hospital, National Cancer Center Hospital East, and Chiba Cancer Center. This study was conducted in accordance with the Declaration of Helsinki.

### Assessment

Treatment response was assessed with CT and, if suitable, MRI. Tumor burden was defined as the total sum of all measured lesions. Besides RECIST version 1.1, we classified responses to PD-1 blockade monotherapies into three groups: nonmixed responders (all metastatic lesions regressing and no presence of recurrences or new lesions), mixed responders (simultaneously regressing and progressing metastatic lesions or with new lesions), and nonmixed nonresponders (progressive metastatic lesions without any sites of tumor regression). Mixed or nonmixed response to the treatment was measured during the first 6-month scans (33).

### IHC

Formalin-fixed, paraffin-embedded (FFPE) sections (3 μm) were deparaffinized with xylene, rehydrated, and subjected to an antigen retrieval process in a microwave oven for 20 minutes. After the inhibition of endogenous peroxidase activity, individual slides were then incubated overnight at 4°C with anti-HLA-I mAb (EMR8–5, Medical & Biological Laboratories Co., Ltd.) and anti-CD8 mAb (human: C8/144B, Dako and mouse: 4SM15, Invitrogen, Thermo Fisher). The slides were then incubated with an EnVision reagent (Dako), and the color reaction was developed in 2% 3,3-diaminobenzidine in 50 mmol/L Tris buffer (pH 7.6) containing 0.3% hydrogen peroxidase. Regarding Supplementary Fig. S1, automated IHC assays that used anti-CD8 mAb (C8/144B, Dako) and anti–PD-L1 mAb (22C3, Dako) were performed using Autostainer Link 48 (Agilent) according to the manufacturer's protocol.

### Multiplexed Fluorescent IHC

Multiplexed fluorescent IHC was performed with direct detection of antigens by primary antibodies from the different species. Anti-CD8 rabbit mAb (SP16, Abcam), and anti–PD-1 mouse mAb (NAT105, Abcam), were used for primary staining. Anti-rabbit IgG Alexa Fluor 555 (Abcam) and anti-mouse IgG Alexa Fluor 488 (Thermo Fisher Scientific) were applied for secondary antibodies. PD-1^+^CD8^+^ T cells were counted in five randomly selected fields using a Leica SP8 confocal microscope (Leica).

### Cell Lines

The establishment of tumor cell lines was performed as previously described (28). Briefly, we cultured 1 × 10^7^ digested tumor cells in RPMI1640, including 10% FBS (FBS; Thermo Fisher Scientific), 100 U/mL penicillin, 100 μg/mL streptomycin, 50 μg/mL gentamicin, and 0.25 μg/mL amphotericin B (Thermo Fisher Scientific). We passaged tumors at approximately 80%–90% confluent, which was used when free of fibroblasts and growing beyond 10 passages.

MC-38 cell line (mouse colon cancer) was purchased from Kerafast. NFAT-luciferase reporter Jurkat (NFAT-Luc-Jurkat) cell line was purchased from InvivoGen. Jurkat cell line was maintained in RPMI1640 medium (Thermo Fisher Scientific) and MC-38 cell line was maintained in DMEM (Thermo Fisher Scientific). Both were supplemented with 10% FBS. All tumor cells were used after confirming that they were negative for *Mycoplasma* using the PCR Mycoplasma Detection Kit (TaKaRa) according to the manufacturer's protocol.

### WES for Human Tumors

WES was performed as previously described (28). Briefly, we isolated genomic DNA from each cell line using a QIAamp DNA Mini Kit (QIAGEN). For enrichment of exonic fragments, the SureSelect Human All Exon Kit v6 (Agilent Technologies) was used. The isolated fragments were sequenced with a HiSeq3000 in massively parallel (Illumina). After masking nucleotides with sequence quality value of less than 20, WES reads were subsequently subjected to alignment to the human reference genome (hg38) using BWA (http://bio-bwa.sourceforge.net/) and Bowtie2 (http://bowtie-bio.sourceforge.net/bowtie2/index.shtml). We identified somatic synonymous and nonsynonymous mutations using our in-house caller and two publicly available mutation callers: Genome Analysis Toolkit (https://gatk.broadinstitute.org/hc/en-us), MuTect2, and VarScan2 (http://varscan.sourceforge.net/). Mutations were excluded if any of the following criteria were met: The total read number was <20, the variant allele frequency in tumor samples was <0.05, the mutant read number in the germline control samples was >2, the mutation was present in only one strand of the genome, or the variant was reported in normal human genomes in either the 1000 Genomes Project dataset (https://www.internationalgenome.org/) or our in-house database. Gene mutations were annotated by SnpEff (https://pcingola.github.io/SnpEff/). We estimated copy number status with our in-house pipeline, in which the logR ratio (LRR) as calculated as follows: (1) SNP positions registered in the 1000 Genomes Project database that were in a homozygous state (VAF ≤ 0.05 or ≥0.95) or a heterozygous state (VAF 0.4–0.6) in the genomes of respective normal samples were selected (2), normal and tumor read depths at the selected position were adjusted based on the G+C percentage of a 100 bp window flanking the position (3), the LRR was calculated as 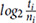, where *n_i_* and *t_i_* are normal and tumor-adjusted depths at position *i*, and (4) each representative LRR was determined as the median of a moving window (1 Mb) centered at position *i*.

### WES and RNA-sequencing for the Mouse Cell Line

WES and RNA-sequencing were performed as previously described (28). Briefly, DNA and RNA were isolated from each cell line using a QIAamp DNA Mini Kit and a RNeasy Mini Kit (QIAGEN), respectively. Genomic DNA was enriched for exonic fragments using the SureSelect mouse (Agilent Technologies). Massively parallel sequencing of isolated fragments was performed with a DNBSEQ-G400 (MGI Tech). After masking nucleotides with sequence quality value of less than 20, WES reads were independently aligned to the mouse reference genome (mm10) using BWA (http://bio-bwa.sourceforge.net/) and Bowtie2 (http://bowtie-bio.sourceforge.net/bowtie2/index.shtml). We identified somatic synonymous and nonsynonymous mutations using our in-house caller and two publicly available mutation callers: Genome Analysis Toolkit (https://gatk.broadinstitute.org/hc/en-us), MuTect2, and VarScan2 (http://varscan.sourceforge.net/). Mutations were excluded if any of the following criteria were met: The total read number was <20, the variant allele frequency in tumor samples was <0.05, or the mutation were detected in only one strand of the genome. Gene mutations were annotated using SnpEff (https://pcingola.github.io/SnpEff/). Poly-A–selected RNA libraries were prepared using the SureSelect Strand-Specific RNA Library Preparation Kit for Illumina (Agilent Technologies). RNA sequencing was performed on an Illumina NextSeq 500 (Ilumina). After sequence acquisition and basic quality confirmation using FastQC (https://www.bioinformatics.babraham.ac.uk/projects/fastqc/), RNA-sequencing reads were aligned to the mm10 mouse genome and quantified with Subread and FeatureCounts (http://subread.sourceforge.net). Genes with low expression were filtered and processed into count-per-million reads by edgeR (https://bioconductor.org/packages/release/bioc/html/edgeR.html).

### Comparison of RNA-sequencing

For comparison of RNA sequencing, fold changes (FC) were calculated such that genes with elevated expression in the #2D10 clone had a log_2_ FC value greater than 0. Expression levels were also compared by a gene-wise exact test under the assumption that RNA-sequencing counts were negative-binomially distributed with a biological coefficient of variation of 0.1 (Supplementary Table S5). Processed data were then underwent gene-set enrichment analysis (GSEA) under default settings against a background of 50 hallmark gene sets curated in MSigDB (https://www.gsea-msigdb.org/gsea/index.jsp).

### Clonal Analysis in Tumor Cells

Copy-number alterations were detected with FACETS (34). The cellular prevalence of clones carrying individual nonsynonymous mutations was determined with PyClone (35). The determined cellular prevalence was used as input, and the phylogenetic relationships of the clones were inferred with LICHeE (36). The results were visualized with the Timescape package in R (R Foundation for statistical computing, Vienna, Austria; ref. 37).

### Neoantigen Prediction

The NetMHCpan algorithm version 4.0 was used to predict the possible neoantigens from the WES data (38, 39). The residues surrounding the amino acids resulting from nonsynonymous mutations were scanned to identify candidate 9-mer peptides that were predicted to bind to the MHC class I alleles of the cells. Strong binding peptides with %Rank ≤0.5 were used for assays (Supplementary Table S6).

### scRNA/TCR-seq

scRNA/TCR-seq was performed as previously described (28). We prepared the libraries using the 10x Single Cell Immune Profiling Solution Kit according to the manufacturer's protocol. Briefly, sorted CD3^+^ T cells were washed and resuspended in PBS with 0.5% FBS, which were subsequently captured in droplets at a targeted cell recovery of <10,000 cells. After reverse transcription and cell barcoding in droplets, the emulsions were broken, and the purified cDNA by Dynabeads MyOne SILANE (Thermo Fisher Scientific) was amplified, which was then used for both 5′ gene expression library construction and TCR enrichment. For construction of gene expression libraries, 2.4–50 ng of amplified cDNA was fragmented, end-repaired, double-sided size-selected with SPRIselect beads (Beckman Coulter), PCR-amplified with sample indexing primers, and again double-sided size-selected with SPRIselect beads. For construction of TCR libraries, we enriched TCR transcripts from 2 μL of amplified cDNA by PCR. Following the enrichment, 5–50 ng of enriched PCR product was fragmented and end-repaired, size-selected with SPRIselect beads, PCR-amplified with sample-indexing primers, and again size-selected with SPRIselect beads. We sequenced the scRNA and scTCR libraries using a HiSeq 3000 instrument to a minimum sequencing depth of 25,000 reads and 5,000 reads per cell, respectively. Sequencing read lengths were adjusted for each library type according to the manufacturer's protocol and reagent version.

### Single-cell Sequencing Data Analysis

The data analysis was performed as previously described (28). Briefly, the reads were processed with CellRanger software (version 6.02). Using UMI count matrices loaded via the Seurat R package (40), cells with a mitochondrial content above 10% and cells with less than 200 or more than 4,000 genes detected were filtered out as dying cells, empty droplets, and doublets, respectively. For normalization, we used the Seurat NormalizeData function, which were subsequently integrated by the IntegrateData function. The dimension was reduced by running PCA and then computing UMAP embeddings using the first 15 components of the PCA for visualization and clustering. Finally, we manually annotated each cluster based on the expression of known marker genes, including *CD3D*, *CD3E*, and *CD2* (T cells); *CD4* and *CCR7* (naive CD4^+^ T cells); *CD8A* and *CCR7* (naive CD8^+^ T cells), *CD4* and *FOXP3* (regulatory T cells); *CD4* and *GZMA* (combined with lack of *CD8A*, cytotoxic CD4^+^ T cells); *CD4*, *CD200,* and *CXCL13* (follicular helper T cells); *CD4* and *CD69* (activated/memory CD4^+^ T cells); *CD8A* and *CD69* (activated CD8^+^ T cells); *CD8A* and *EOMES* (memory CD8^+^ T cells); and *CD8A, PDCD1,* and *HAVCR2* (exhausted CD8^+^ T cells) (28).

We aligned TCR reads to the GRCh38 reference genome, and used cellranger vdj (10x Genomics, version 4.0.0) for consensus TCR annotation according to the manufacturer's protocol. As the matching to public bulk TCR datasets is usually performed at the TCRβ level, cells with no reconstructed TCRα clonotype were kept, whereas cells with no TCRβ clonotype were not retained for following analysis. For cells with two or more clonotypes, we used the clonotype with the highest number of UMIs. If the number of UMIs in the second clonotype was more than half of the top clonotype, the cells were labeled as ambiguous.

### Virus Production and Transfection

The procedures of virus production and transfection were performed as previously described (28). Briefly, Each TCRα- and β-transduced pMSCV vector was created by VectorBuilder, which were transfected into packaging cells with a pVSV-G vector (TaKaRa) using Lipofectamine 3000 Reagent (Thermo Fisher Scientific). After 48 hours, we concentrated the supernatant, which was subsequently transfected into the NFAT-Luc-Jurkat cell line.

### Luciferase Reporter Assay Using Peptides

We selected TCRs with commercially available antibodies from skewed exhausted T-cell clonotypes in LN1 and LN2 (#1-#4) (Supplementary Table S7). The procedure was performed as previously described (28). Briefly, we confirmed each TCR-transduced NFAT-Jurkat cell line using anti-TCR mAb, which was subsequently cocultured with autologous cells after the pulse of each peptide. After 24 hours, luciferase activity was analyzed using QUANTI-Luc (InvivoGen) according to the manufacturer's protocol. We calculated the fold change in each NFAT-Luc-Jurkat cell line without each peptide pulse (DMSO). *In vitro* experiments were performed in triplicate. We selected control clonotype #0 from minor clones in the TME that were frequently found in MHC-matched Adaptive Biotechnologies public PBL datasets (41).

### *In Vivo* Animal Experiments

*In vivo* experiment was performed as previously described (28). Briefly, we purchased female C57BL/6J mice (6–8 weeks old) from SLC Japan, and RIKEN BRC provided C57BL/6J-Prkdc<scid>/Rbrc mice (B6 SCID; RBRC01346) through the National BioResource Project of the MEXT/AMED, Japan. Cells (1 × 10^6^) were subcutaneously inoculated, and tumor volume was measured twice a week. For tumor growth curves, we used the means of the long and short diameters, and grouped mice when the tumor volume reached approximately 100 mm^3^. Thereafter, anti-PD-1 mAb (200 μg/mouse) or control mAb was administered intraperitoneally three times every 3 days. Tumors were harvested at baseline (approximately 100 mm^3^) to collect TILs for evaluation. When different MC-38 clones were used *in vivo*, each clone was subcutaneously inoculated at different sites in the same mouse. Rat anti-mouse PD-1 mAb (RMP1–14) and control rat IgG2a mAb (RTK2758) were obtained from BioLegend. We performed i*n vivo* experiments at least twice. All mice were maintained under specific pathogen-free conditions in the animal facility of the Institute of Biophysics. Mouse experiments were approved by the Animal Committee for Animal Experimentation of the Chiba Cancer Center. All *in vivo* experiments met the U.S. Public Health Service Policy on Humane Care and Use of Laboratory Animals.

### Bulk TCR Sequencing and Data Analysis

TCR sequencing for clinical samples and mouse samples was performed on extracted RNA with Oncomine TCR Beta-SR Assay (Thermo Fisher Scientific) and SMARTer Mouse TCRa/b Profiling Kit (TaKaRa), respectively. Human TCR libraries were sequenced using Ion Torrent S5 (Thermo Fisher Scientific) and then analyzed with Ion Reporter Software (Thermo Fisher Scientific). In addition, we used the Illumina MiSeq platform (Illumina) and a 2 × 300 bp paired-end kit for the sequencing of mouse TCR libraries, and then analyzed the data with algorithms described previously (42).

### Flow Cytometry Analyses

Flow cytometry assays were performed as described (43). Briefly, cells were washed with PBS containing 2% FBS and subjected to staining with surface antibodies. Intracellular staining was performed with specific antibodies and the FOXP3/Transcription Factor Staining Buffer Set (Thermo Fisher Scientific) according to the manufacturer's protocol. For intracellular cytokine staining, GolgiPlug reagent (BD Biosciences) was added for the last 4 hours of culture. Samples were assessed with a BD FACSVerse instrument (BD Biosciences) and FlowJo software (BD Biosciences). The staining antibodies were diluted following the manufacturer's instructions. Supplementary Table S8 summarizes the antibodies used in the flow cytometry analyses.

### Statistical Analyses

GraphPad Prism 8 (GraphPad Software) or R version 4.0.2 was used for statistical analyses. We compared patients’ characteristics among groups using the Fisher exact test. The comparison of continuous variables among groups were performed using the *t* test or one-way ANOVA. The comparisons among tumor volume curves were performed using two-way ANOVA. Bonferroni corrections were applied for multiple testing. Progression-free survival (PFS) and overall survival (OS) were defined as the time from the initiation of PD-1 blockade therapies until the first observation of disease progression or death from any cause and until death from any cause, respectively. We analyzed PFS and OS using the Kaplan–Meier method and compared among groups using a log-rank test. To estimate HRs, we also used a Cox proportional hazards model. *P*-values of <0.05 were considered statistically significant.

### Data Availability

The data discussed in this publication have been deposited in Japanese Genotype-Phenotype Archive (JGA). Accession numbers are JGAS000285 and JGAD000391.

## Results

### A Patient had Different Tumor Cell Clones and TME in Two Lymph Node Metastatic Lesions Next to Each Other

A male in his late 60s with recurrent facial superficial spreading melanoma received anti-PD-1 mAb as 1st line therapy. Before the treatment, he received surgical resection of occipital lymph node metastases as diagnosis, and the remaining pulmonary metastases responded to anti–PD-1 mAb (Fig. 1A).

While the occipital LN metastasis was resected *en bloc*, two separate metastatic lesions were found to be separated by connective tissue (Fig. 1A). We obtained two samples from each lesion next to each other (LN1 and LN2). While they were located next to each other and both had CD8^+^ T-cell infiltration, LN1 had a significantly higher frequency of PD-1^+^CD8^+^ T cells in the TME than LN2 (Fig. 1B). We next performed WES for these two lesions. LN1 had *BRAF* fusion but LN2 did not, and there was a significant difference in tumor cell clones between LN1 and LN2 (Fig. 1C and D). These findings suggest that there could be both tumor and immune cell heterogeneities between LN1 and LN2.

### Skewed Exhausted T Cell Clones Were Considerably Found in the TME From LN1 but not from LN2

**FIGURE 2:**
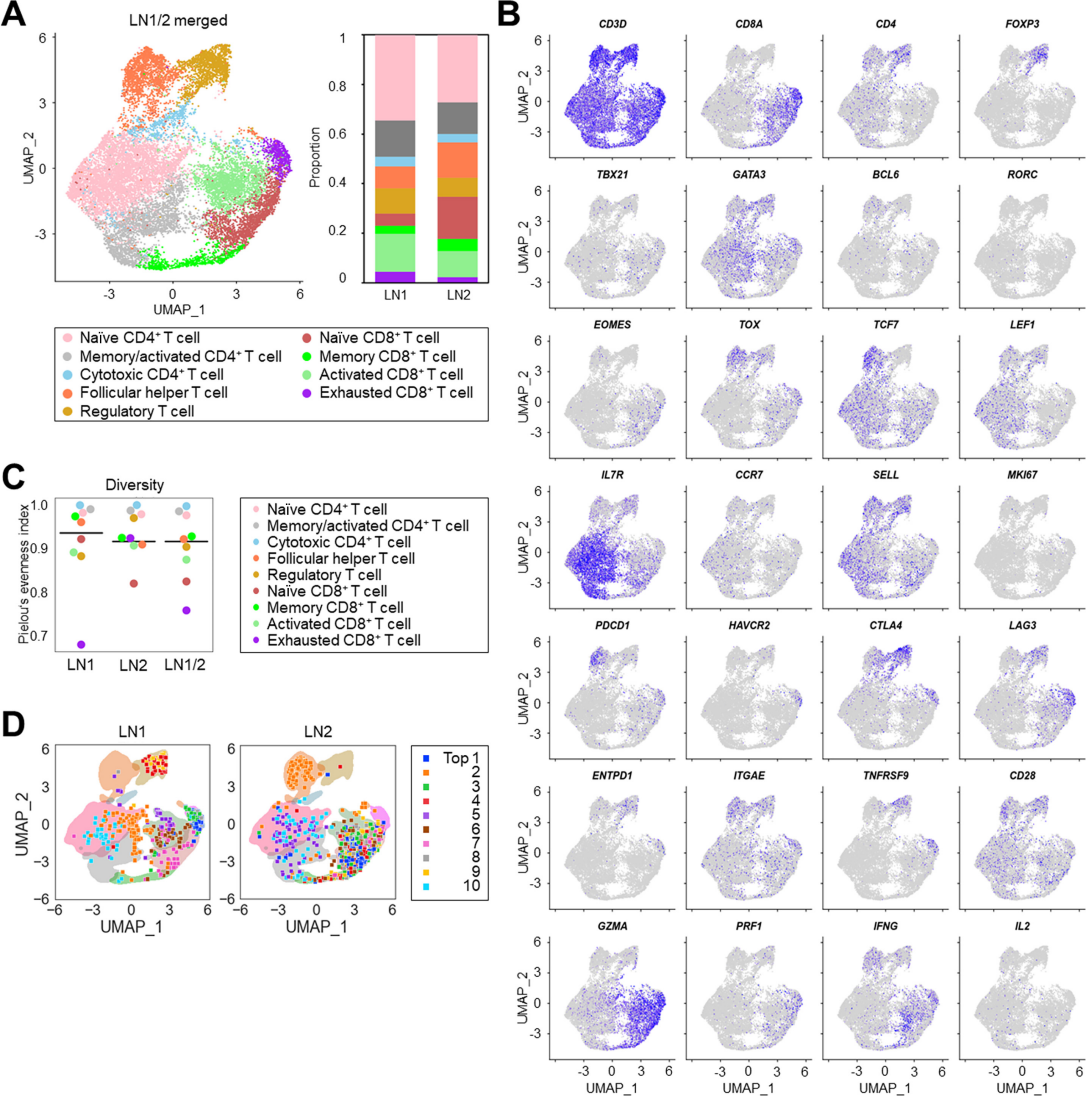
LN1, but not LN2, had skewed exhausted T-cell clones in the TME. **A,** T-cell clustering. We digested LN1 and LN2 to extract TILs. Single-cell sequencing was performed for sorted CD3^+^ T cells from the extracted TILs. The data of two TIL samples were merged and then clustered based on gene expression. UMAP figure (left) and the summary (right) are presented. **B,** Representative gene expression in each cluster. Representative genes that are generally used for annotation are shown. **C,** T-cell clonotype diversity. Pielou's evenness indexes of each sample and each cluster are calculated and summarized. Bars indicate the indexes of all clusters. **D,** Top 10 clonotype distributions from each sample. The distribution of the top 10 clonotypes from each TIL sample in the UMAP figure is presented.

We analyzed the tumor-infiltrating T cells from LN1 and LN2 using droplet-based 5′ single-cell RNA sequencing and single-cell T cell receptor (TCR) sequencing. In total, we obtained paired TCR sequences in 18,637 out of 32,674 T cells (70.5%, Supplementary Table S9). As previously reported, we classified T cells into 9 clusters based on gene expression profiling (Fig. 2A and B; Supplementary Fig. S2A; refs. 28, 44). LN1 had a considerable population of exhausted T-cell cluster characterized by exhaust signature genes such as *PDCD1*, *TNFRSF9*, *ENTPD1*, and *ITGAE,* whereas LN2 had few (Fig. 2A and B; Supplementary Fig. S2B). High PD-1 expression in CD8^+^ T cells, particularly in exhausted T cells, was also observed in LN1 (Supplementary Fig. S2C). We next focused on T-cell clones since we previously reported that skewed exhausted T cell clones in the TME were tumor-specific (28, 45–47). Accordingly, the TCR of the exhausted cluster was skewed compared with other clusters, particularly in LN1 (Fig. 2C). Furthermore, when we plotted the top 10 clonotypes from each sample into the UMAP figure, some of the clonotypes from LN1 were classified into the exhausted cluster, whereas those from LN2 were not (Fig. 2D), suggesting that tumor-specific exhausted T-cell clones were skewed in LN1 but not in LN2. A regulatory T-cell cluster was observed with greater TCR skewing in LN1 compared with LN2 (Fig. 2A–D; Supplementary Fig. S2A and B). These results indicate heterogeneity in T-cell clones, such as the presence of tumor-specific exhausted T-cell clones, between LN1 and LN2.

### An Overlapped Exhausted T-Cell Clone Responded to an Overlapped Neoantigen

**FIGURE 3:**
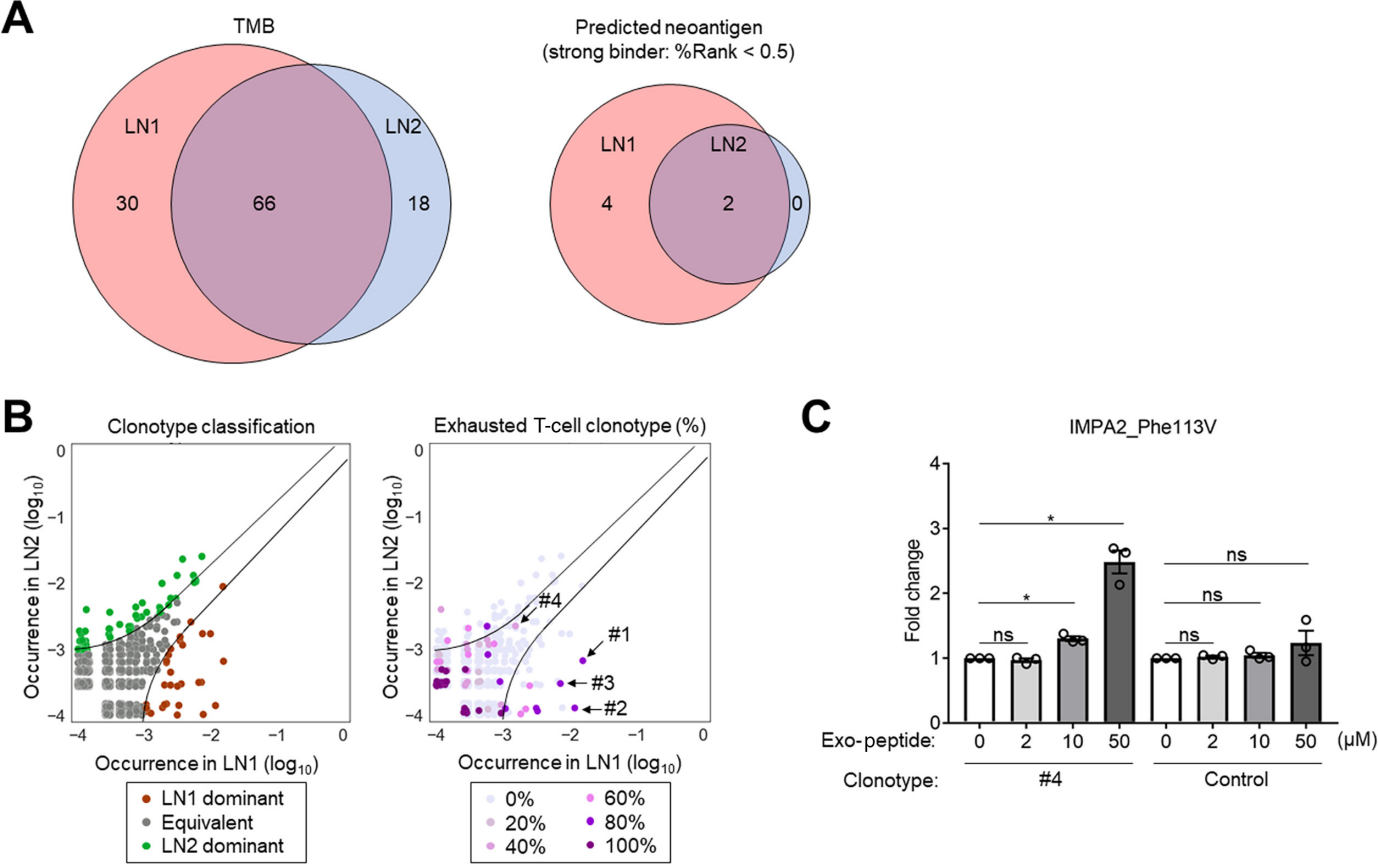
An overlapped exhausted T cell clonotype responded to an overlapped neoantigen. **A,** Venn diagrams of nonsynonymous somatic mutations (TMB) and predicted neoantigens. WES for LN1 and LN2 was performed. We used the NetMHCpan algorithm version 4.0 in order to predict the neoantigen candidates from the WES data (38, 39). The numbers of nonsynonymous somatic mutations (left) and predicted neoantigens with strong binding (right) are shown. **B,** Clonotype in LN1 and LN2. We merged the clonotypes of LN1 with LN2, which were grouped into LN1 dominant, equivalent, or LN2 dominant clonotypes according to their frequencies. The UMAP figures of clonotype groups (left) and exhausted T cell frequency (right) are shown. Arrows, tested clonotypes in Supplementary Table S7. **C,** Peptide assay. After mutated IMPA2 peptide pulse, TCR-transduced NFAT-Luc-Jurkat cell lines were cocultured with autologous cells for 24 hours. Thereafter, luciferase activity was analyzed. The fold change in each NFAT-Luc-Jurkat cell line with no peptide pulse (DMSO) is presented. All *in vitro* experiments were performed in triplicate, and we show the means and SEMs. To calculate statistical significance in **C,** one-way ANOVA with the Bonferroni correction were used. *, *P* < 0.05; ns, not significant.

Both tumor and T-cell heterogeneities between LN1 and LN2 led us to analyze neoantigen heterogeneities in tumor cells. Nonsynonymous somatic mutations and predicted neoantigens are summarized in Fig. 3A. While nonsynonymous somatic mutations were considerably found in both LN1 and LN2 and considerable numbers were overlapped, many of the predicted neoantigen with strong binding (%Rank < 0.5) were found in LN1 (Fig. 3A; Supplementary Table S6). On the other hand, there was no predicted neoantigen with strong binding found in only LN2 (Fig. 3A). We next merged TCR clonotypes between LN1 and LN2 (Fig. 3B). There were some skewed exhausted T-cell clonotypes in LN1 dominant clonotypes, whereas we found few exhausted T-cell clonotypes in LN2 dominant clonotypes (9/33 vs. 4/50, *P* < 0.01; Fig. 3B).

We created each TCR-transduced NFAT-Luc-Jurkat cell line, and investigated luciferase activity using each peptide from predicted neoantigens. We selected TCRs with commercially available antibodies from skewed exhausted T-cell clonotypes in LN1 and LN2 (#1-#4) and neoantigens with strong binding (Supplementary Tables S6 and S7). The responses of some selected TCRs to the autologous cell line was confirmed in our previous study (28), and TCR expression was confirmed with flow cytometry (Supplementary Fig. S3A). Among the clonotypes and neoantigens that we tested, an overlapped exhausted T-cell clonotype (#4) responded to an overlapped neoantigen (IMPA2_F113V, DGTCNFVHRVPTVAVSIGF; Fig. 3C; Supplementary Fig. S3B).

### A Mouse Cell Line has Different Tumor Cell Clones with Different TME Related to a Mixed Response to PD-1 Blockade

**FIGURE 4:**
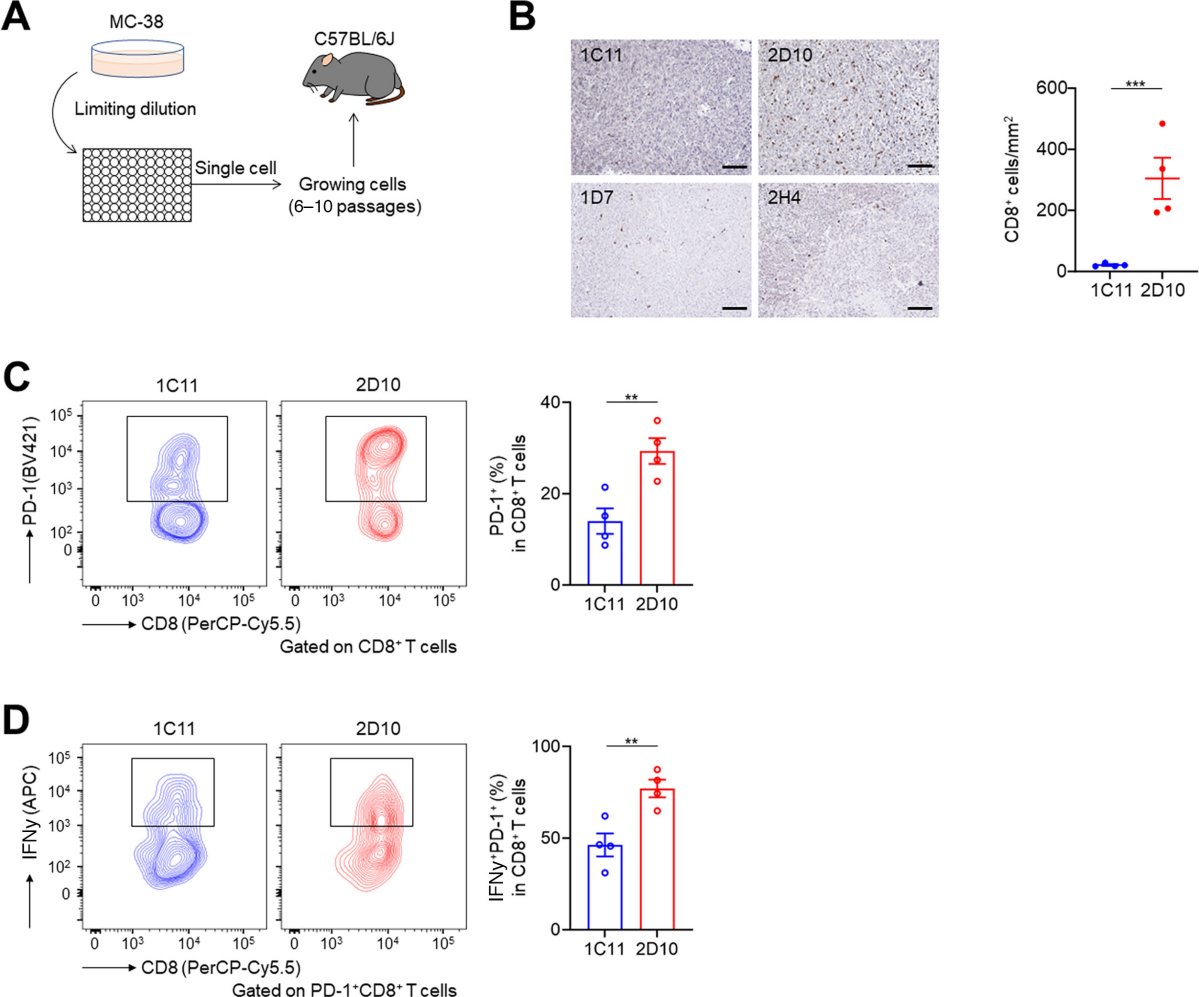
Mouse tumors from different clones had different TME. **A,** Graphic of the experimental schema of *in vivo* experiments. We created several single tumor cell clones from the same mouse tumor cell line and injected them subcutaneously. **B,** IHC for CD8. FFPE sections (3 μm) of tumor samples from each clone were stained. Representative CD8 staining is presented. **C** and **D,** PD-1 expression by CD8^+^ T cells (**C**) and IFNɤ^+^PD-1^+^CD8^+^ T cells (**D**) in the TME. Representative flow cytometry staining (left) and the summaries (right) are shown. All *in vivo* experiments were performed in duplicate and produced similar results. The means and SEMs are shown. T-tests were used to calculate statistical significance in **B**, **C**, and **D**. **, *P* < 0.01; ***, *P* < 0.001.

**FIGURE 5:**
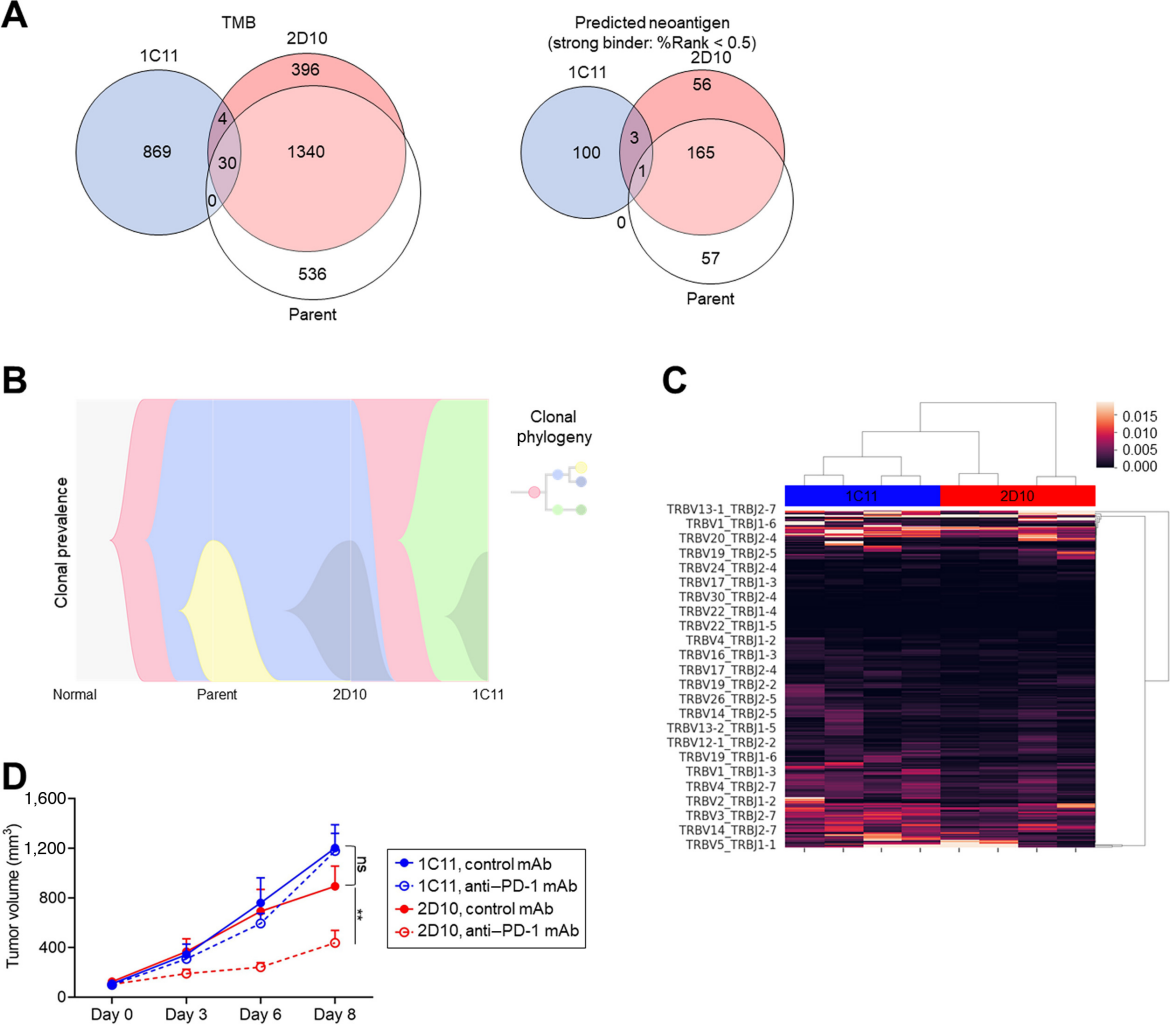
Mouse tumors from different clones exhibited mixed responses to PD-1 blockade. **A,** Venn diagrams of nonsynonymous somatic mutations (TMB) and predicted neoantigens. WES for MC-38 (parent), MC-38 #1C11, and MC-38#2D10 was performed. We used the NetMHCpan algorithm version 4.0 in order to predict the neoantigen candidates from the WES data (38, 39). The numbers of nonsynonymous somatic mutations (left) and predicted neoantigens with strong binding (right) are shown. **B,** Clonal evolution of tumor cells. The cellular prevalence of clones with individual nonsynonymous mutations in parental MC-38, #1C11, and #2D10 was determined with PyClone. As input, the determined cellular prevalence was used, and the clonal phylogenetic relationships were inferred using LICHeE. **C,** TCR clustering. Bulk RNA were extracted from tumors and sequenced for TCR and the results were clustered. **D,** Efficacy of PD-1 blockade in a mouse model. Cells (1 × 10^6^) were subcutaneously inoculated in immunocompetent wild-type mice, and tumor volume was measured twice a week. We grouped mice when the tumor volume reached approximately 100 mm^3^ (*n* = 6 per group). Afterward, anti-PD-1 mAb or control mAb was administered intraperitoneally three times every 3 days. All *in vivo* experiments were performed in duplicate with similar results. We show the means and SEMs. To calculate statistical significance in **D,** two-way ANOVA with Bonferroni correction were used. **, *P* < 0.01; ns, not significant.

Our human clinical sample data showing heterogeneous tumor cell clones with heterogeneous exhausted CD8^+^ T-cell clonal infiltration suggest that the efficacy of immunotherapies can be different among heterogeneous lesions in the same patient. Thus, we next created several single tumor cell clones from the same mouse tumor cell line (Fig. 4A). Briefly, single clones were cultured in 96-well plates at limiting dilution and then gradually expanded to 24-well plates and 10-cm dishes. The number of passages was between 6 and 10. Cellular morphology remained constant between passages (Supplementary Fig. S4A). IHC for CD8 demonstrated that CD8^+^ T-cell infiltration differed among the clones (Fig. 4B). On the basis of CD8^+^ T-cell infiltration, we selected two clones (#1C11, CD8 lowest; #2D10, CD8 highest) and investigated the detailed TME, subcutaneously injecting each clone at different sites of the same mice (Supplementary Fig. S5). TIL analyses demonstrated that PD-1^+^CD8^+^ T cells and IFNγ-producing PD-1^+^CD8^+^ T cells were frequently found in the TME from #2D10 but were less frequently found from #1C11 even in the same mice (Fig. 4C and D). On the other hand, MHC-I expression analyzed by flow cytometry tended to be higher in #1C11 than in #2D10 with or without IFNγ stimulation *in vitro* (Supplementary Fig. S4B), and GSEA from RNA-sequencing *in vitro* demonstrated that immune-response-related hallmarks such as IFNɑ or IFNγ response were suppressed in #2D10 (Supplementary Fig. S4C). For example, #1C11 had higher *Cd274* (encoding PD-L1) expression levels compared with #2D10 (log_2_ FC = −1.27; Supplementary Table S5). Thus, evaluating differences in immune cell infiltration between the clones was challenging using MHC-I expression and RNA-sequencing data. In contrast, WES indicated that both TMB and the number of predicted neoantigens were more frequently found in #2D10 when compared with #1C11 (Fig. 5A). In addition, most somatic mutations and neoantigens were not shared among them (Fig. 5A). Accordingly, substantial tumor clone heterogeneity was observed, with clone #1C11 found to be particularly rare among heterogeneous parental MC-38 cells (Fig. 5B). Furthermore, a well-known SNV rs13480628 *Jmid1c* in MC-38 was found in both clones, indicating these cells were derived from parental MC-38 cells (48). TCR sequencing also indicated different T-cell clones and small shared TCR clonotypes between tumors, as seen for somatic mutations and neoantigens (Fig. 5C; Supplementary Fig. S6A). On the other hand, many somatic mutations and neoantigens were shared between the parental MC-38 and #2D10 (Fig. 5A). Anti–PD-1 mAb exhibited efficacy against parental MC-38 and #2D10 tumors but not against #1C11 tumors even in the same mice (Fig. 5D; Supplementary Figs. S5 and S6B). In immunocompetent mice, #1C11 tumors tended to grow faster than #2D10 tumors; however, the difference was not statistically significant (Fig. 5D). No significant difference in tumor growth or PD-1 blockade–mediated efficacy was observed between #1C11 and #2D10 tumors in immunodeficient mice (Supplementary Fig. S6C). These findings suggest that heterogeneous tumor and T-cell clones can induce a heterogeneous mixed response to cancer immunotherapies.

### Patients with a Mixed Response to Immunotherapies Have a Poorer Prognosis Compared to Nonmixed Responders

**FIGURE 6:**
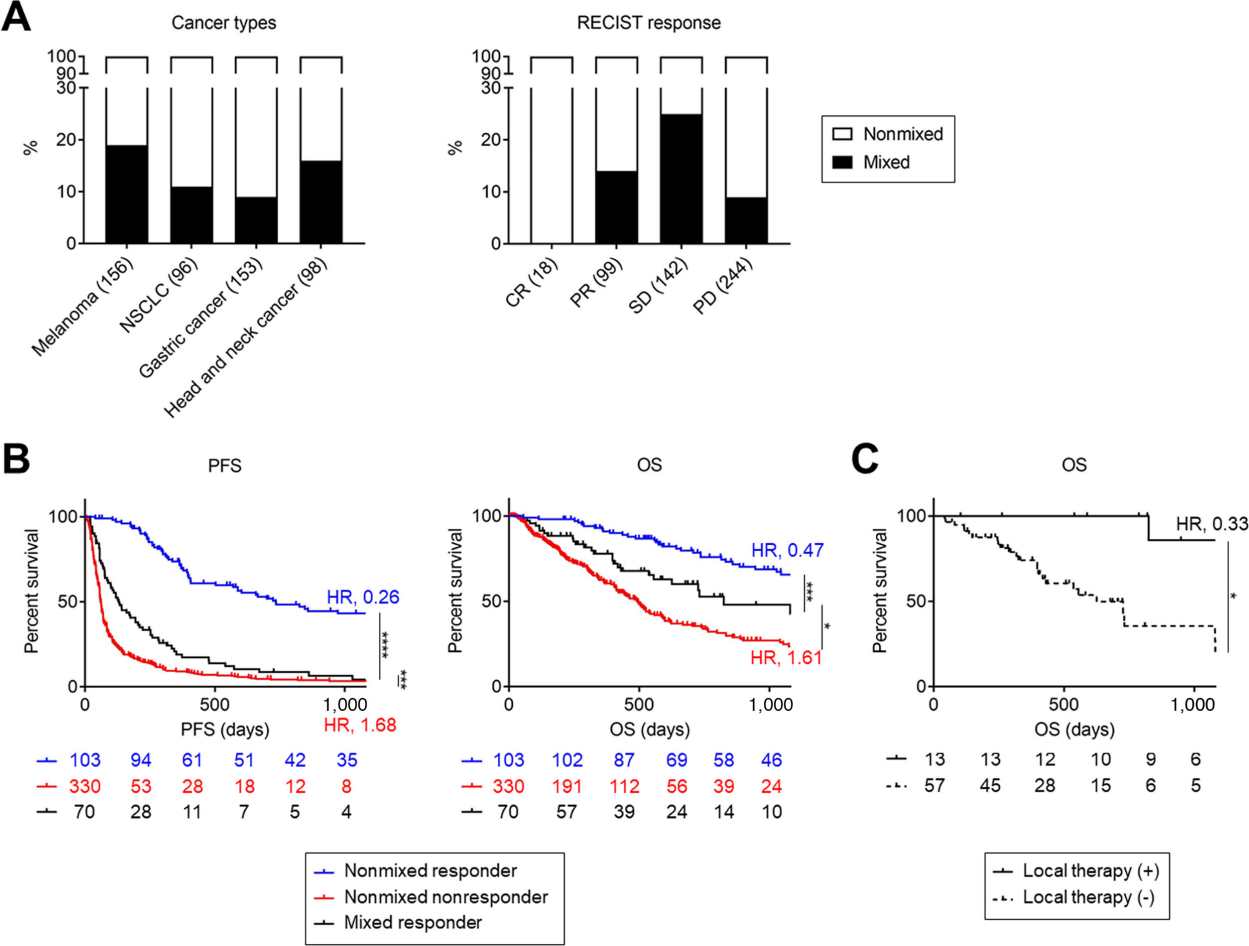
Mixed responders to immunotherapies had a poor prognosis compared with nonmixed responders in various cancer types. **A,** The frequencies of mixed responses in various cancer types. We investigated the clinical data in several cancer types, including melanoma, NSCLC, gastric cancer, and head and neck cancer, treated with PD-1 blockade monotherapies. A mixed response was defined as previously reported (33). The frequencies according to cancer types (left) or RECIST response (right) are presented. **B** and **C,** Survival curves. We compared a prognosis in all patients among nonmixed responders, nonmixed nonresponders, and mixed responders. A prognosis in mixed responders was also compared according to local therapies. PFS and OS were defined as the time from the initiation of PD-1 blockade therapies until the first observation of disease progression or death from any cause and the time from the initiation of PD-1 blockade therapies until death from any cause, respectively. PFS (left) and OS (right) in all patients according to responses (**B**) and OS in mixed responders according to local therapies (**C**) are presented. PFS and OS were analyzed using the Kaplan–Meier method and compared among groups using a log-rank test. *, *P* < 0.05; **, *P* < 0.01; ****, *P* < 0.0001.

Finally, we investigated clinical data about the mixed response to immunotherapies in several cancer types, including melanoma, NSCLC, gastric cancer, and head and neck cancer, who received PD-1 blockade monotherapies. Clinical data of 503 patients are summarized in Supplementary Tables S1–4. A mixed response was defined as previously reported (33). Briefly, we classified responses to PD-1 blockade monotherapies into three groups: nonmixed responders (all metastatic lesions regressing and no presence of recurrences or new lesions); mixed responders (simultaneous regression and progression of metastatic lesions or new lesions); and nonmixed nonresponders (progressive metastatic lesions without tumor regression at any site). Mixed or nonmixed responses to treatment was determined from initial 6-month scans (33). There were 70 mixed responders in our cohort (total, 13.9%; melanoma, 18.6%; head and neck cancer, 16.3%; NSCLC, 11.5%; gastric cancer, 9.2%; Fig. 6A and Supplementary Tables S1–4). Mixed responses were mainly observed in patients with RECIST SD, especially in melanoma and head and neck cancer (Fig. 6A and Supplementary Tables S1–4). We compared the prognosis among patients with nonmixed responses, nonmixed non-responses, and mixed responses. Both PFS and OS of mixed responders were shorter than those with nonmixed responders (Fig. 6B). A gastric cancer patient experienced tumor response to anti-PD-1 mAb followed by disease progression in only perigastric LN metastasis (Supplementary Fig. S1A and B). Pathologically, HLA class I (HLA-I) positive and negative tumor cells were mixed in the primary lesion with heterogeneous CD8^+^ T-cell infiltration, whereas the progressed LN lesion had only HLA-I negative tumor cells with low CD8^+^ T-cell infiltration (Supplementary Fig. S1B). Accordingly, there were significant differences in both tumor cell clones and TCRs between the primary and LN lesions (Supplementary Fig. S1C and D).

Thirteen of 70 mixed responders (18.6%) received local therapies (radiotherapy, 10; surgical resection, 2; arterial injection chemotherapy for liver metastasis, 1), and these patients tended to have longer OS than the others among mixed responders (Fig. 6C). Heterogeneities in both tumor and immune cells contributed to mixed responses to immunotherapies, leading to a poor prognosis. Local therapies for resistant lesions may improve prognosis in such patients.

## Discussion

The kinetics and heterogeneity of immunotherapy responses are insufficiently evaluated by RECIST version 1.1. Recently, Rauwerdink and colleagues found that mixed responders in the melanoma cohort were enriched for RECIST SD with an intermediate survival outcome, and show that these responses are dynamic and can evolve (33). We also found that a heterogeneous mixed response to immunotherapies in various types of cancer is not uncommon with an intermediate prognosis. In addition, we demonstrated that mixed responses to immunotherapies could be related to both tumor and immune cells, especially tumor-specific T cells, heterogeneities from both human clinical sample and mouse model data. Thus, the heterogeneity in tumor tissue including not only tumor but also immune cells could be an important barrier for cancer immunotherapies, leading to a poor prognosis compared with nonmixed responders.

From single-cell sequencing for clinical samples, tumor-infiltrating exhausted T-cell clones were different between LN1 and LN2. In particular, exhausted T-cell clones, which reportedly directly attack tumor cells (28, 45–47), were more enriched in LN1 than in LN2. Tumor cell clones were also different between LN1 and LN2 from WES. Especially, predicted neoantigens were enriched in LN1 compared with LN2, which is consistent with enrichment of exhausted T-cell clones in LN1. From these findings, the difference in tumor cell clones including neoantigens could induce tumor-specific exhausted T-cell clonal difference. In addition, skewed regulatory T-cell clones were also enriched in LN1, which can be recruited as part of inflammatory responses (49, 50). Thus, tumor-specific exhausted T cells that attack tumor cells directly in LN1 may recruit skewed regulatory T-cell clones. Thus, different tumor cell clones may be associated with different T-cell clonal infiltrations. In a mouse model to study tumor cell clones originating from the same mouse cell line, #2D10 tumors had high PD-1^+^CD8^+^ T-cell infiltration and responded to PD-1 blockade, whereas #1C11 tumors failed to respond to PD-1 blockade and displayed low PD-1^+^CD8^+^ T-cell infiltration. These can reflect mixed responses in clinical settings. Therefore, tumor and immune cell heterogeneities, which can be related to each other, can cause mixed responses to immunotherapies, leading to a poor prognosis compared with nonmixed responders.

We analyzed more than 500 patients across different cancer types, showing that the frequencies of mixed response were varied among cancer types. While the frequency in melanoma is similar to that in a previous retrospective study (33), there has been no comparison among various cancer types. Thus, this present study is the first report to compare these frequencies. Although this study includes several biases, the frequencies of mixed responses in melanoma or head and neck cancer patients seemed higher, indicating that these cancer types could have more heterogeneities. The patient analyzed in detail in our study received surgical resection for both LN1 and LN2, and the remaining lesions responded to ICI, although LN2 could be resistant. Considering his clinical course, mixed responders can get clinical benefits from local therapies for resistant lesions such as surgical resection and/or radiotherapy. Indeed, locally treated mixed responders had longer OS in our cohort, and a previous study has also shown a similar tendency (33). In addition, other studies have shown that local therapies in patients with heterogeneous responses, such as oligometastatic progression and mixed response to immunotherapies, can render patients disease-free (51, 52). In our cohort, local therapies mainly consisted of radiotherapies (10/13), and we previously reported increased efficacy of radiotherapy after PD-1 blockade therapies due to enhanced antitumor immunity (53). From these findings, local therapies for remaining lesions, including radiotherapy, should be considered for mixed responders to immunotherapies.

This study has some limitations. First, we could analyze heterogeneities of T-cell clones in detail from just one case. Thus, we created several mouse tumor cell clones with different TME from the same cell line to validate human clinical sample data. Although not all mouse experiments were performed using different sites of the same mice and we did not analyze neoantigen-specific T cells, one clone responded to PD-1 blockade with high PD-1^+^CD8^+^ T-cell infiltration while the other failed to respond to blockade and had low PD-1^+^CD8^+^ T-cell infiltration. These findings may indicate mixed responses are to be expected in clinical settings. Although a mixed response was not observed in our clinical case after surgical resection, there were significant differences in both tumor and immune cell clones between the lesions. Significant differences in both tumor and T-cell clones between primary and LN lesions in our clinical case of gastric cancer, with disease progression observed only in LN lesions with the loss of HLA-I. Second, we could prove just one neoantigen to which an exhausted CD8^+^ T-cell clonotype responded. Many previous studies, including our study, have demonstrated that not all predicted neoantigen are truly neoantigens (28) and prediction models have limitations. Although the difference among tumor cell clones, especially in neoantigens, could induce the difference in exhausted CD8^+^ T-cell clonal infiltration, the proved neoantigen was overlapped between LN1 and LN2. Otherwise, an exhausted T-cell clonotype that responded to this overlapped neoantigen was also overlapped between LN1 and LN2, which is reasonable for our hypothesis. Third, our mouse model of mixed response did not completely reflect the changes seen in LN1 and LN2. The mouse model rather reflects inflamed (#2D10) vs. non-inflamed TME (#1C11), whereas the clinical cases of LN1 and LN2 both had similar levels of CD8^+^ T-cell infiltration. However, the mouse model partially reflected clinical observations regarding exhausted CD8^+^ T-cell infiltration. Fourth, we retrospectively analyzed approximately 500 patients treated with PD-1 blockade monotherapies, and the clinical characteristics varied. In addition, the number of locally treated mixed responders is tiny. Thus, more extensive prospective analyses should be performed to confirm our findings.

In summary, we analyzed two samples that were closely located to each other with WES and single-cell sequencing. WES for tumors and single-cell sequencing for the TME showed heterogeneities of both tumor cell and exhausted T-cell clones. We also showed that tumor and T-cell heterogeneities could induce mixed responses to immunotherapies using a mouse model. Clinically, patients with mixed responses to immunotherapies had a poorer prognosis than non-mixed responders in various cancer types. These findings suggest that mixed response to immunotherapies can be related to both tumor and immune cell heterogeneities, leading to a poor prognosis, and that such resistant lesions should be locally treated. Because there are several limitations in this study, further basic and clinical researches are required.

## Supplementary Material

Supplementary Figure S1 Clinical course of a gastric cancer patient, IHC, and sequencing data.

Supplementary Figure S2 Additional analyses of scRNA-seq.

Supplementary Figure S3 Peptide assay.

Supplementary Figure S4 Additional in vitro data using the MC-38 clones.

Supplementary Figure S5 Representative photos of mice injected at different sites.

Supplementary Figure S6 Additional in vivo data using MC-38 cells.

Supplementary Table S1 Melanoma patient characteristics.

Supplementary Table S2 NSCLC patient characteristics.

Supplementary Table S3 Gastric cancer patient characteristics.

Supplementary Table S4 Head and neck cancer patient characteristics.

Supplementary Table S5 Comparison of RNA-seq.

Supplementary Table S6. Predicted neoantigen candidates with strong binding (%Rank < 0.5).

Supplementary Table S7 Tested clonotypes.

Supplementary Table S8 Summary of antibodies used in flow cytometry analyses.

Supplementary Table S9 Sequencing summary.
