# Supplementary material for: Mixed Response to Cancer Immunotherapy is Driven by Intratumor Heterogeneity and Differential Interlesion Immune Infiltration
Source: Cancer Res Commun. 2022 Jul 28;2(7):739–53. doi: 10.1158/2767-9764.CRC-22-0050 (PMC10010332; doi:10.1158/2767-9764.CRC-22-0050)
Supplement: Supplementary Figure S1 — Clinical course of a gastric cancer patient, IHC, and sequencing data. [file crc-22-0050-s01.docx]

**Supplementary Figure S1. Clinical course of a gastric cancer patient, IHC, and sequencing data.**

**
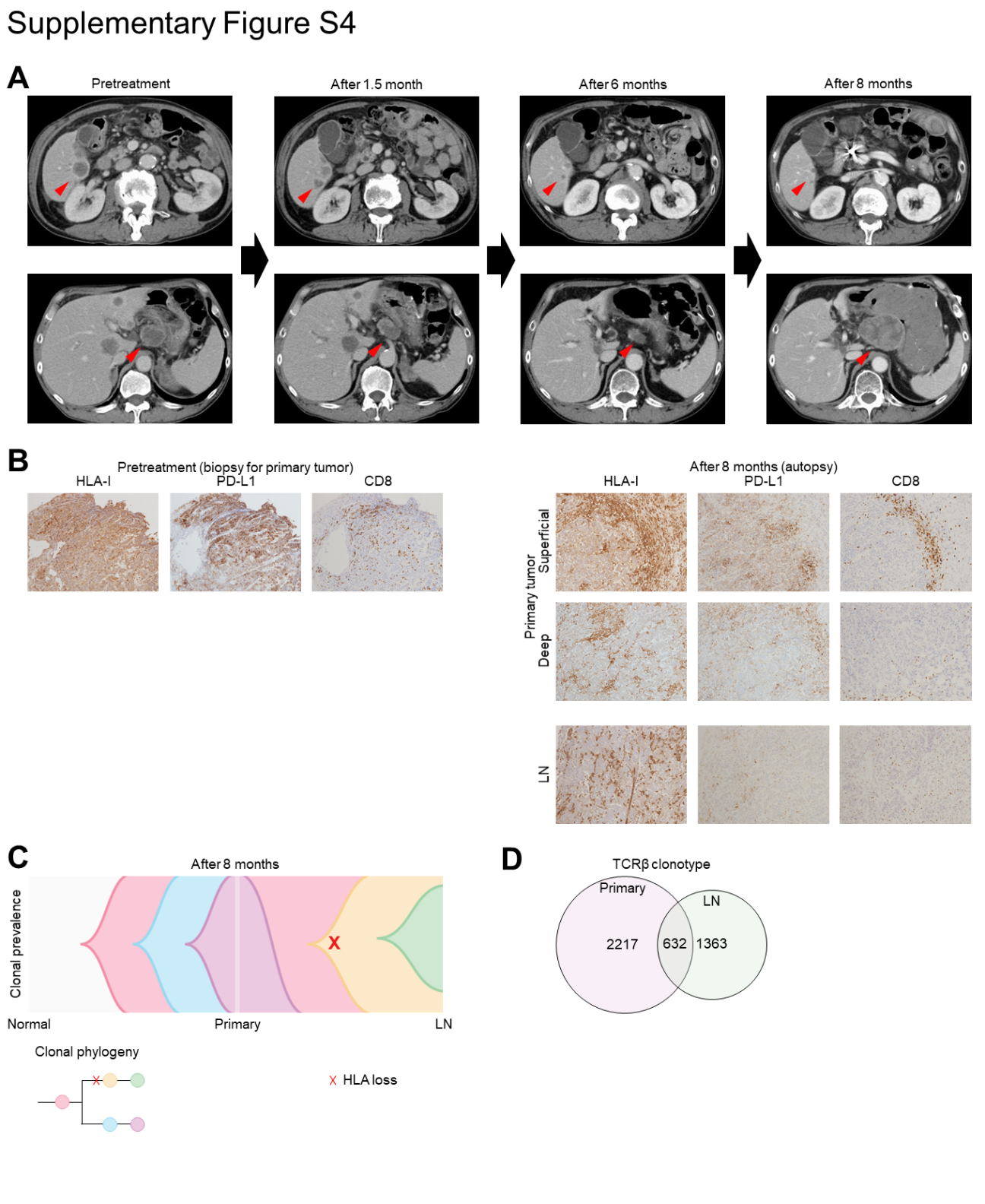
 A,** Computed topographies of a gastric cancer patient. A male in his middle 60s with advanced gastric cancer received anti-PD-1 mAb as the third-line therapy. He achieved partial response initially, but perigastric LN metastasis progressed rapidly after 8 months. He died because of massive gastric bleeding by the arterial rupture with this lesion. Computed topographies at each point are presented. Red arrows, metastatic lesions.

**B,** IHC. Pretreatment biopsy and autopsy samples after 8 months of treatment were analyzed. FFPE sections (3 µm) were used for IHC with HLA-I, PD-L1, and CD8.

**C,** Clonal evolution of tumor cells. The cellular prevalence of clones carrying individual nonsynonymous mutations in each lesion was determined using PyClone. The determined cellular prevalence was used as input, and the phylogenetic relationships of clones were inferred with LICHeE.

**D,** Venn diagram of TCRβ clonotype. Bulk RNA were extracted from tumors and sequenced for TCRβ. Shared and unshared clonotypes between primary and LN lesions are presented.
