# Supplementary material for: Mixed Response to Cancer Immunotherapy is Driven by Intratumor Heterogeneity and Differential Interlesion Immune Infiltration
Source: Cancer Res Commun. 2022 Jul 28;2(7):739–53. doi: 10.1158/2767-9764.CRC-22-0050 (PMC10010332; doi:10.1158/2767-9764.CRC-22-0050)
Supplement: Supplementary Figure S2 — Additional analyses of scRNA-seq. [file crc-22-0050-s02.docx]

**Supplementary Figure S2. Additional analyses of scRNA-seq.**


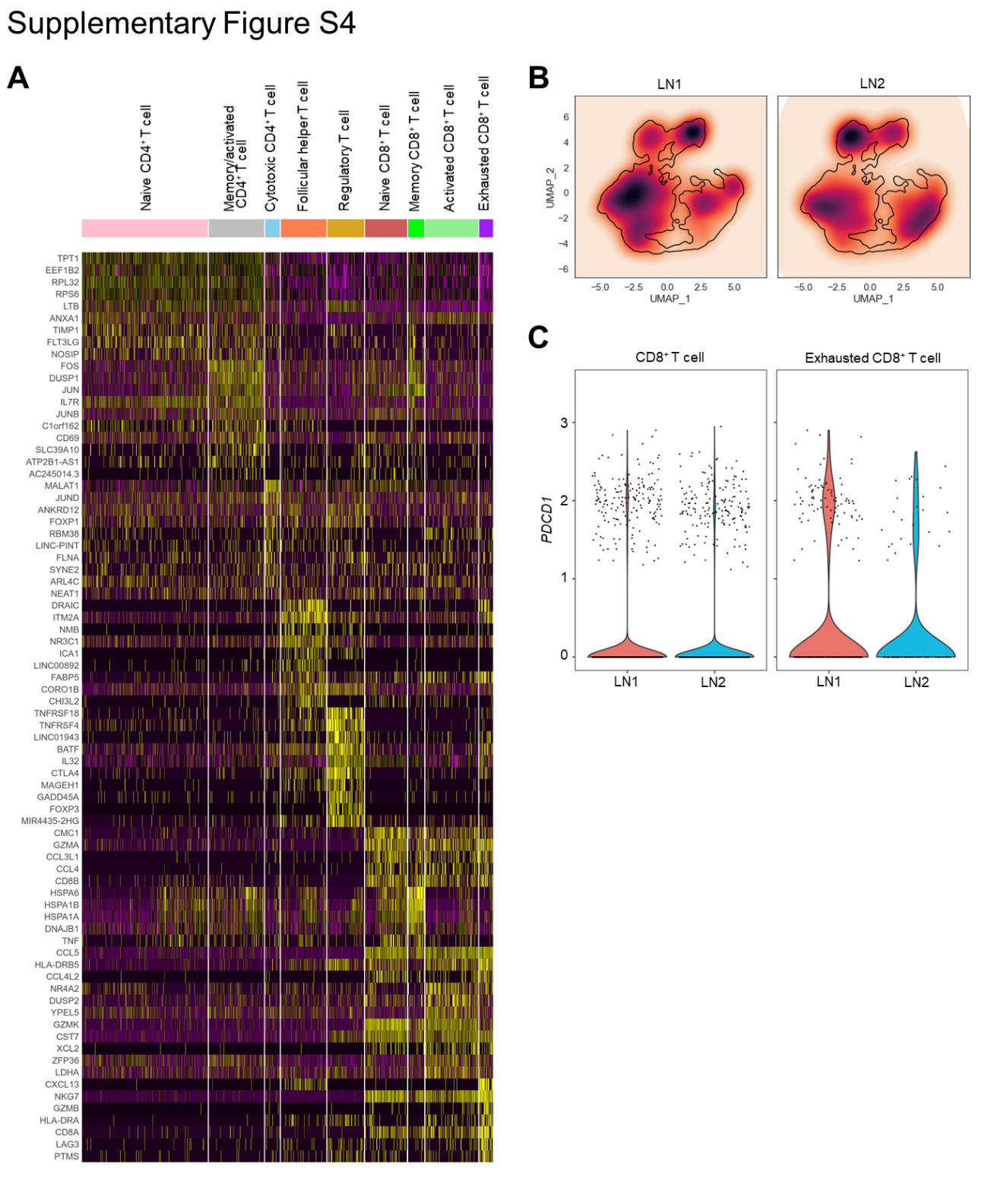


**A,** Heatmap of the normalized RNA expression values of the top differentially expressed genes for each cluster. LN1 and LN2 were digested to extract TILs. Sorted CD3^+^ T cells from TILs were analyzed with single-cell sequencing. Merged data of two TIL samples were clustered using gene expression. Differentially expressed genes were calculated as described, and the top 10 genes (ranked by fold-change and limited to genes detected in at least 5% of cells and with an adjusted *p*-value of 0.05) were plotted. **B,** Clustering of T cells. Separated contour plots of each LN are shown. **C,** *PDCD1* expression in CD8^+^ T cell clusters. *PDCD1* expressions in total CD8^+^ T cell and exhausted CD8^+^ T cell clusters are shown.
