# Supplementary material for: Mixed Response to Cancer Immunotherapy is Driven by Intratumor Heterogeneity and Differential Interlesion Immune Infiltration
Source: Cancer Res Commun. 2022 Jul 28;2(7):739–53. doi: 10.1158/2767-9764.CRC-22-0050 (PMC10010332; doi:10.1158/2767-9764.CRC-22-0050)
Supplement: Supplementary Figure S3 — Peptide assay. [file crc-22-0050-s03.docx]

**Supplementary Figure S3. Peptide assay.**

**
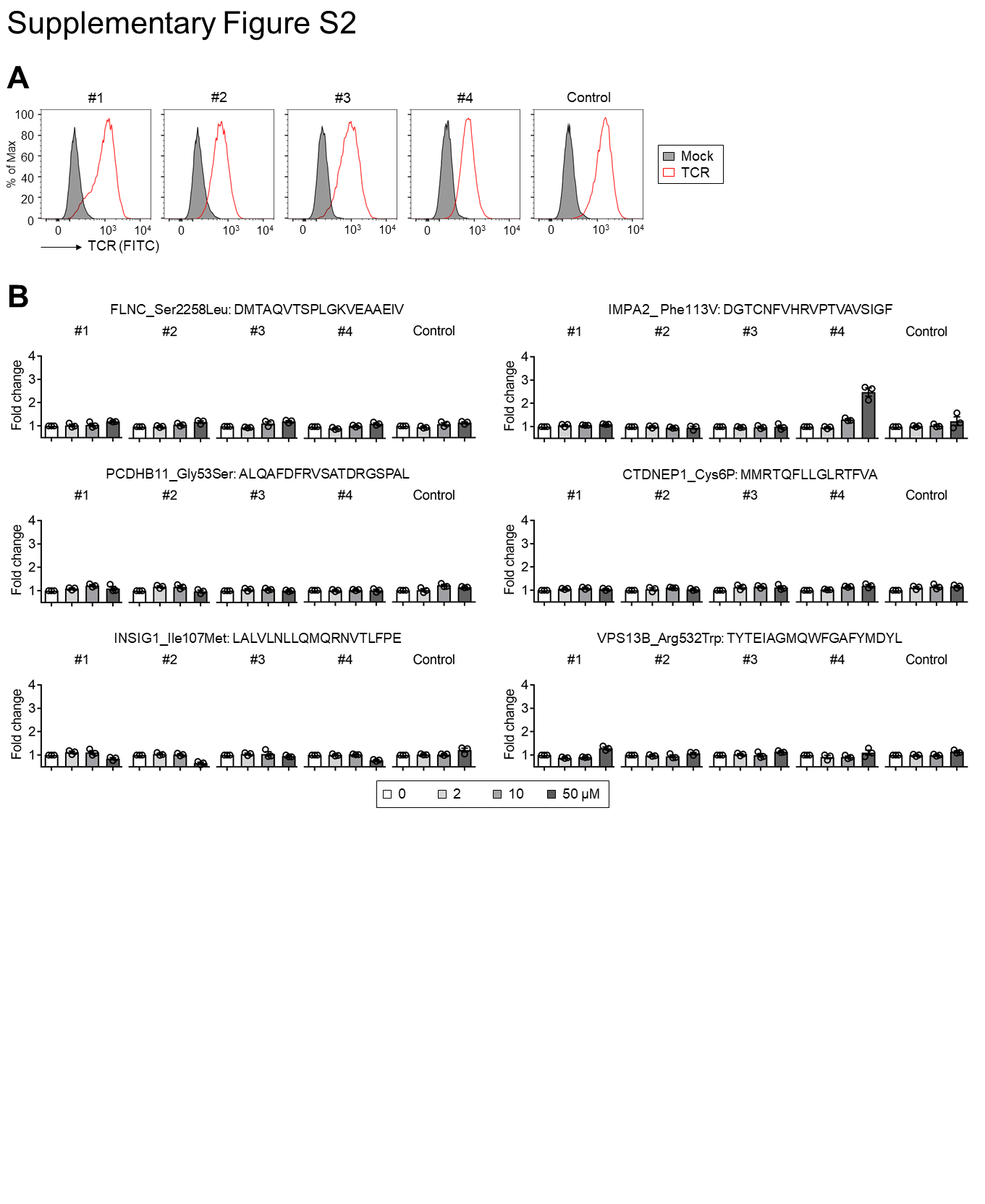
A,** TCR expression. The top skewed TCRs against which antibodies were commercially available were selected among exhausted T cell clonotypes (#1~#4). TCR transduction was confirmed in NFAT-Jurkat cells using flow cytometry after sorting. Representative flow cytometry staining is presented. **B,** TCR-transduced NFAT-Jurkat cell lines were cocultured with autologous cells after each peptide pulse. Luciferase activity was analyzed after 24 hours of coculture. We compared with non-pulsed data for statistical analyses. The fold change in each NFAT-Jurkat cell line without each peptide pulse was calculated, and is shown.

All *in vitro* experiments were performed in triplicate, and the means and SEMs are shown.
