# Supplementary material for: Mixed Response to Cancer Immunotherapy is Driven by Intratumor Heterogeneity and Differential Interlesion Immune Infiltration
Source: Cancer Res Commun. 2022 Jul 28;2(7):739–53. doi: 10.1158/2767-9764.CRC-22-0050 (PMC10010332; doi:10.1158/2767-9764.CRC-22-0050)
Supplement: Supplementary Figure S4 — Additional in vitro data using the MC-38 clones. [file crc-22-0050-s04.docx]

**Supplementary Figure S4. Additional *in vitro* data using the MC-38 clones.**

**
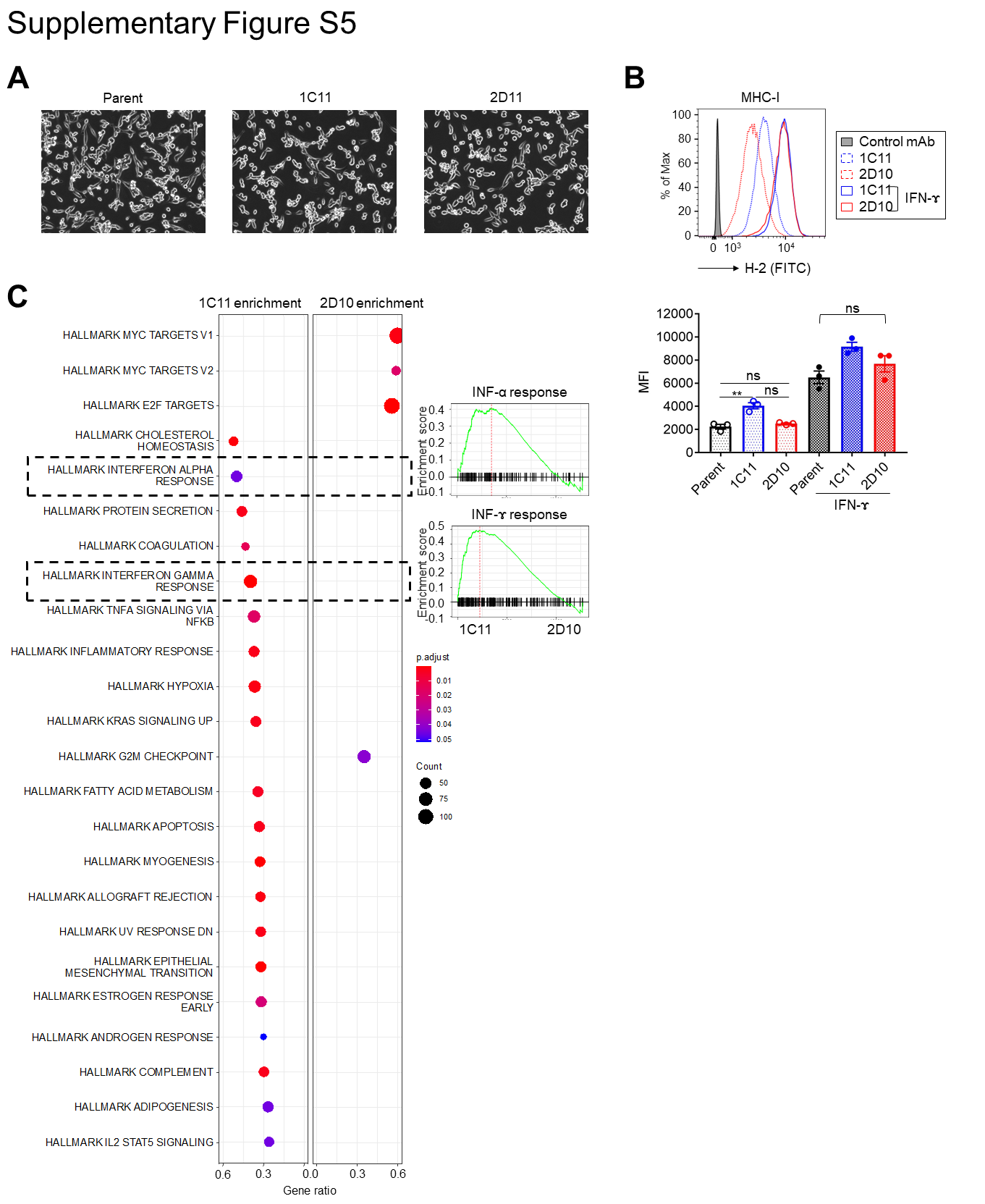
**

**A,** Morphology of clones #1C11 and #2D10. Representative phase contrast microscopy images are presented. **B,** MHC-I expression. MHC-I expression in each cell line was analyzed using flow cytometry. Representative flow cytometry staining (top) and respective summaries (bottom) are shown. *In vitro* experiments were performed in triplicate, with means and SEMs shown. **C,** GSEA. RNA-sequencing for #1C11 and #2D10 was performed *in vitro* and data were compared with GSEA. Top 20 hallmarks (left) and representative immune-related signatures (right) are presented.

One-way ANOVA with Bonferroni correction was used in **B**. **p < 0.01; ns, not significant.
