# Supplementary material for: Mixed Response to Cancer Immunotherapy is Driven by Intratumor Heterogeneity and Differential Interlesion Immune Infiltration
Source: Cancer Res Commun. 2022 Jul 28;2(7):739–53. doi: 10.1158/2767-9764.CRC-22-0050 (PMC10010332; doi:10.1158/2767-9764.CRC-22-0050)
Supplement: Supplementary Figure S5 — Representative photos of mice injected at different sites. [file crc-22-0050-s05.docx]

**Supplementary Figure S5.** **Representative photos of mice injected at different sites.**


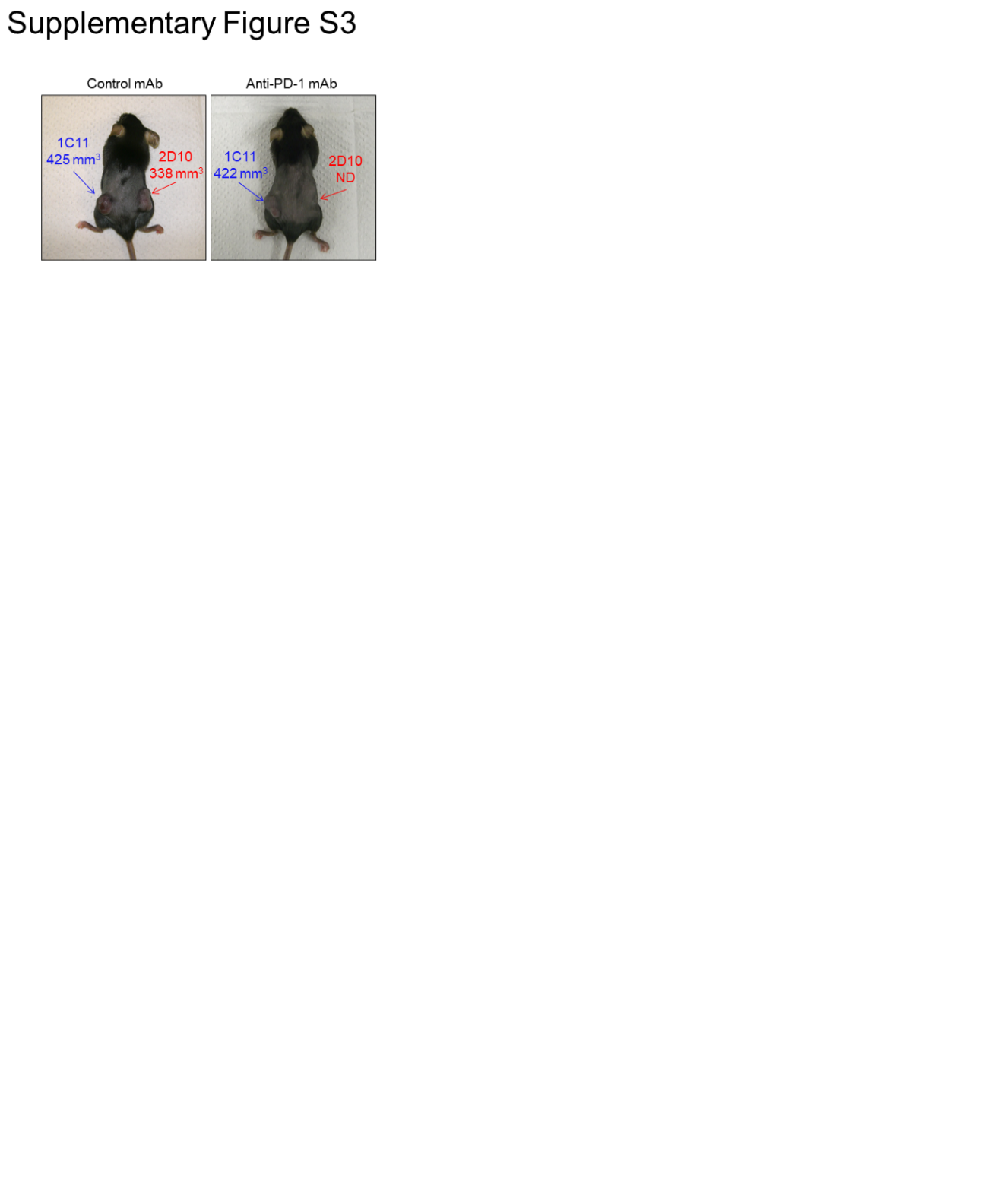
Cells (1 × 10^6^) were subcutaneously injected in different sides of the same immunocompetent wild-type mice (left, #1C11; right, #2D10). Tumor volumes were measured twice a week. Mice were dividing into groups when the tumor volume reached approximately 100 mm^3^, after which anti-PD-1 mAb, or control mAb were intraperitoneally administered three times at intervals of 3 days. ND, not detected.
