# Supplementary material for: Mixed Response to Cancer Immunotherapy is Driven by Intratumor Heterogeneity and Differential Interlesion Immune Infiltration
Source: Cancer Res Commun. 2022 Jul 28;2(7):739–53. doi: 10.1158/2767-9764.CRC-22-0050 (PMC10010332; doi:10.1158/2767-9764.CRC-22-0050)
Supplement: Supplementary Figure S6 — Additional in vivo data using MC-38 cells. [file crc-22-0050-s06.docx]

**Supplementary Figure S6. Additional *in vivo* data using MC-38 cells.**

**
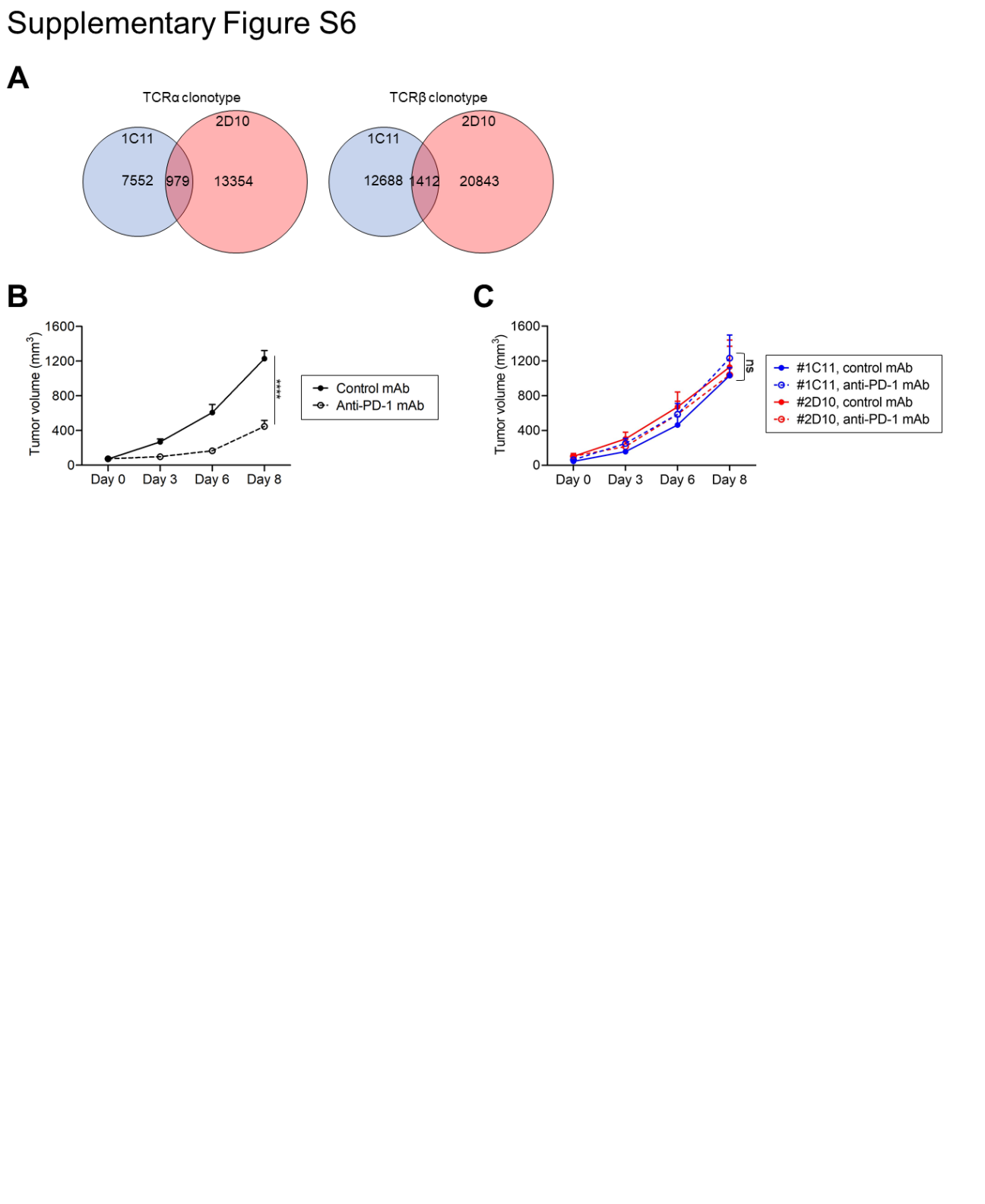
A,** Venn diagrams of TCR clonotypes. Bulk RNA was extracted from tumors and sequenced for TCRα/β. Shared and unshared clonotypes between all #1C11 and #2D10 tumors are presented. **B,** Efficacy of PD-1 blockade against parental MC-38 tumors in immunocompetent mice. Cells (1 × 10^6^) were subcutaneously injected into immunocompetent wild-type mice. Tumor volume was measured twice a week. Mice were dividing into groups when the tumor volume reached approximately 100 mm^3^ (n = 6 per group), after which anti-PD-1 mAb, or control mAb were intraperitoneally administered three times at intervals of 3 days. **C,** Efficacy of PD-1 blockade in immunodeficient mice. Cells (1 × 10^6^) were injected subcutaneously in B6 SCID mice, and tumor volume was monitored twice a week. Mice were grouped when the tumor volume reached approximately 100 mm^3^ (n = 5 per group), after which anti-PD-1 mAb or control mAb was administered intraperitoneally three times at intervals of 3 days.

All *in vivo* experiments were performed in duplicate and produced similar results. Means and SEMs are shown. To calculate statistical significance, two-way ANOVA was used in **B**, with the Bonferroni correction used in **C**. ****p < 0.0001; ns, not significant.
