# Supplementary material for: Mixed Response to Cancer Immunotherapy is Driven by Intratumor Heterogeneity and Differential Interlesion Immune Infiltration
Source: Cancer Res Commun. 2022 Jul 28;2(7):739–53. doi: 10.1158/2767-9764.CRC-22-0050 (PMC10010332; doi:10.1158/2767-9764.CRC-22-0050)
Supplement: Supplementary Table S1 — Melanoma patient characteristics. [file crc-22-0050-s07.docx]

**Supplementary Table S1. Melanoma patient characteristics.**

| Features | Non-mixed (127) | Mixed (29) | *p* |
| --- | --- | --- | --- |
| **Age, years** [median] (range) | 69 (22–89) | 73 (43–86) | 0.12 |
| **Sex** (male/female) | 70/57 | 14/15 | 0.54 |
| **Performance status** (0 or 1/2–) | 120/7 | 29/0 | 0.35 |
| **Type**  (cutaneous or primary unknown/ acral or mucosal/others/ND) | 42/72/12/1 | 9/17/1/2 | > 0.99¶ |
| **Stage** (-III/IV) | 34/93 | 4/25 | 0.16 |
| **ICI treatment line**  (1st line/2nd line-) | 120/7 | 25/4 | 0.12 |
| ***BRAF* status**  (mutated/wild-type/NE) | 104/18/5 | 18/7/4 | 0.14 |
| **Response to PD-1 blockade**  (RECIST CR/PR/SD/PD) | 7/30/31/59 | 0/7/17/5 | < 0.001¶¶ |

ND, not determined; NE, not evaluated; ¶cutaneous or primary unknown vs. acral or mucosal; ¶¶SD vs. CR, PR, or PD.
