# Supplementary material for: Mixed Response to Cancer Immunotherapy is Driven by Intratumor Heterogeneity and Differential Interlesion Immune Infiltration
Source: Cancer Res Commun. 2022 Jul 28;2(7):739–53. doi: 10.1158/2767-9764.CRC-22-0050 (PMC10010332; doi:10.1158/2767-9764.CRC-22-0050)
Supplement: Supplementary Table S2 — NSCLC patient characteristics. [file crc-22-0050-s08.docx]

**Supplementary Table S2. NSCLC patient characteristics.**

| Features | Non-mixed (85) | Mixed (11) | *p* |
| --- | --- | --- | --- |
| **Age, years** [median] (range) | 68 (40–94) | 64.5 (57–77) | 0.97 |
| **Sex** (male/female) | 59/26 | 9/2 | 0.50 |
| **Performance status** (0 or 1/2–) | 82/3 | 11/0 | > 0.99 |
| **Type**  (adenocacinoma/squamous cell carcinoma/others) | 67/14/4 | 9/2/0 | > 0.99¶ |
| **Stage** (recurrence/III-IV) | 19/66 | 5/6 | 0.14 |
| **ICI treatment line**  (1st line/2nd line-) | 20/65 | 4/7 | 0.46 |
| ***EGFR* or *ALK* status**  (mutated/wild-type/NE) | 18/66/1 | 0/11/0 | 0.12 |
| **Response to PD-1 blockade**  (RECIST CR/PR/SD/PD) | 1/16/35/33 | 0/2/7/2 | 0.20¶¶ |

NE, not evaluated;¶adenocarcinoma vs. squamous cell carcinoma; ¶¶SD vs. CR, PR, or PD.
