# Supplementary material for: Mixed Response to Cancer Immunotherapy is Driven by Intratumor Heterogeneity and Differential Interlesion Immune Infiltration
Source: Cancer Res Commun. 2022 Jul 28;2(7):739–53. doi: 10.1158/2767-9764.CRC-22-0050 (PMC10010332; doi:10.1158/2767-9764.CRC-22-0050)
Supplement: Supplementary Table S3 — Gastric cancer patient characteristics. [file crc-22-0050-s09.docx]

**Supplementary Table S3. Gastric cancer patient characteristics.**

| Features | Non-mixed (139) | Mixed (14) | *p* |
| --- | --- | --- | --- |
| **Age, years** [median] (range) | 69 (25–86) | 66.5 (35–85) | 0.89 |
| **Sex** (male/female) | 100/39 | 10/4 | 0.10 |
| **Performance status** (0 or 1/2–) | 124/15 | 14/0 | 0.36 |
| **Type**  (intestinal/diffuse) | 60/79 | 7/7 | 0.78 |
| **HER2 status**  (positive/negative) | 24/115 | 3/11 | 0.71 |
| **Response to PD-1 blockade**  (RECIST CR/PR/SD/PD) | 2/20/28/89 | 0/2/3/9 | > 0.99¶ |

All patients had stage IV gastric cancer and received 2nd line or later ICI. ¶SD vs. CR, PR, or PD.
