# Supplementary material for: Mixed Response to Cancer Immunotherapy is Driven by Intratumor Heterogeneity and Differential Interlesion Immune Infiltration
Source: Cancer Res Commun. 2022 Jul 28;2(7):739–53. doi: 10.1158/2767-9764.CRC-22-0050 (PMC10010332; doi:10.1158/2767-9764.CRC-22-0050)
Supplement: Supplementary Table S4 — Head and neck cancer patient characteristics. [file crc-22-0050-s10.docx]

**Supplementary Table S4. Head and neck cancer patient characteristics.**

| Features | Non-mixed (82) | Mixed (16) | *p* |
| --- | --- | --- | --- |
| **Age, years** [median] (range) | 63 (32–77) | 62.5 (43–74) | 0.53 |
| **Sex** (male/female) | 60/22 | 9/7 | 0.23 |
| **Performance status** (0 or 1/2–) | 79/3 | 15/1 | 0.52 |
| **Type**  (oropharyngeal/hypopharyngeal/oral/larynx) | 28/22/27/5 | 5/3/6/2 | > 0.99¶ |
| **Response to PD-1 blockade**  (RECIST CR/PR/SD/PD) | 8/19/13/42 | 0/3/8/5 | 0.0054¶¶ |

All patients had stage IV recurrent or metastatic head and neck cancer and received ICI after platinum-based therapies. ¶oropharyngeal vs. others; ¶¶SD vs. CR, PR, or PD.
