# Supplementary material for: Mixed Response to Cancer Immunotherapy is Driven by Intratumor Heterogeneity and Differential Interlesion Immune Infiltration
Source: Cancer Res Commun. 2022 Jul 28;2(7):739–53. doi: 10.1158/2767-9764.CRC-22-0050 (PMC10010332; doi:10.1158/2767-9764.CRC-22-0050)
Supplement: Supplementary Table S6. — Predicted neoantigen candidates with strong binding (%Rank < 0.5). [file crc-22-0050-s12.docx]

**Supplementary Table S6.** **Predicted neoantigen candidates with strong binding (%Rank < 0.5).**

| Rank | HLA | Identity | Score | Affinity | %Rank | Sample |
| --- | --- | --- | --- | --- | --- | --- |
| **1** | C*01:02 | FLNC_ Ser2258L | 0.39806 | 673.7 | 0.0219 | LN1/2 |
| **2** | C*01:02 | IMPA2_ Phe113V | 0.312861 | 1693.7 | 0.1167 | LN1/2 |
| **3** | C*01:02 | PCDHB11_ Gly53 | 0.308807 | 1769.7 | 0.1252 | LN1 |
| **4** | A*33:03 | CTDNEP1_ Cys6P | 0.600277 | 75.6 | 0.2957 | LN1 |
| **5** | A*24:02 | INSIG1_ Ile107 | 0.527021 | 166.9 | 0.3145 | LN1 |
| **6** | B*15:01 | VPS13B_ Arg532 | 0.620455 | 60.7 | 0.3241 | LN1 |
