# Supplementary material for: Mixed Response to Cancer Immunotherapy is Driven by Intratumor Heterogeneity and Differential Interlesion Immune Infiltration
Source: Cancer Res Commun. 2022 Jul 28;2(7):739–53. doi: 10.1158/2767-9764.CRC-22-0050 (PMC10010332; doi:10.1158/2767-9764.CRC-22-0050)
Supplement: Supplementary Table S7 — Tested clonotypes. [file crc-22-0050-s13.docx]

**Supplementary Table S7. Tested clonotypes**

| Clonotype | V | D | J | CDR3 |
| --- | --- | --- | --- | --- |
| **#1**  **(LN1 dominant)** | TRAV8-4 |  | TRAJ34 | CAVTGLYNTDKLIF |
|  | TRBV6-5 | TRBD1 | TRBJ1-2 | CASSYSGQGGNYGYTF |
| **#2**  **(LN1 dominant)** | TRAV3 |  | TRAJ22 | CAVSLLSSGSARQLTF |
|  | TRBV19 |  | TRBJ2-7 | CASSILGHSYEQYF |
| **#3**  **(LN1 dominant)** | TRAV19 |  | TRAJ33 | CALSEAVDSNYQLIW |
|  | TRBV5-1 | TRBD1 | TRBJ2-1 | CASSFAGGYEQFF |
| **#4**  **(Equivalent)** | TRAV19 |  | TRAJ54 | CALGGGQGAQKLVF |
|  | TRBV12-3 |  | TRBJ2-1 | CASARQPGTSSYNEQFF |
| **Control** | TRAV39 |  | TRAJ47 | CAVDREYGNKLVF |
|  | TRBV5-1 |  | TRBJ1-1 | CASSLGTPNTEAFF |
