# Supplementary material for: Mixed Response to Cancer Immunotherapy is Driven by Intratumor Heterogeneity and Differential Interlesion Immune Infiltration
Source: Cancer Res Commun. 2022 Jul 28;2(7):739–53. doi: 10.1158/2767-9764.CRC-22-0050 (PMC10010332; doi:10.1158/2767-9764.CRC-22-0050)
Supplement: Supplementary Table S8 — Summary of antibodies used in flow cytometry analyses. [file crc-22-0050-s14.docx]

**Supplementary Table S8. Summary of antibodies used in flow cytometry analyses.**

| Molecule | Tag | Clone | Company |
| --- | --- | --- | --- |
| **Mouse CD3** | V500 | 500A2 | BD Biosciences |
| **Mouse CD8** | PerCP-Cy5.5 | 53-6.7 | Biolegend |
| **Mouse PD-1** | BV421 | EH12.2H7 | Biolegend |
| **Mouse IFN-γ** | APC | XMG1.2 | BD Biosciences |
| **Mouse H-2** | FITC | M1/42 | Biolegend |
| **Human TCR Vβ6.5** | FITC | IMMU 222 | Beckman Coulter |
| **Human TCR Vβ19** | FITC | E17.5F3.15.13 | Beckman Coulter |
| **Human TCR Vβ5.1** | FITC | IMMU 157 | Beckman Coulter |
| **Human TCR Vβ12.3** | FITC | 56C5.2 | Beckman Coulter |
