# Supplementary material for: Mixed Response to Cancer Immunotherapy is Driven by Intratumor Heterogeneity and Differential Interlesion Immune Infiltration
Source: Cancer Res Commun. 2022 Jul 28;2(7):739–53. doi: 10.1158/2767-9764.CRC-22-0050 (PMC10010332; doi:10.1158/2767-9764.CRC-22-0050)
Supplement: Supplementary Table S9 — Sequencing summary. [file crc-22-0050-s15.docx]

**Supplementary Table S9.** **Sequencing summary.**

| Sample | Read number | | Cell number | | Median genes/cell | Clonotype number |
| --- | --- | --- | --- | --- | --- | --- |
|  | **RNA** | **TCR** | **RNA** | **TCR** |  |  |
| **LN1** | 308,519,225 | 304,877,362 | 9,999 | 6,786 | 665 | 4,011 |
| **LN2** | 309,943,894 | 351,958,068 | 8,638 | 6,354 | 695 | 4,027 |
| **Mean** | 309,231,560 | 328,417,715 | 9,319 | 6,570 | 680 | 4,019 |
